# Smartphone applications for physical activity and sedentary behaviour change in people with cardiovascular disease: A systematic review and meta-analysis

**DOI:** 10.1371/journal.pone.0258460

**Published:** 2021-10-11

**Authors:** Kacie Patterson, Rachel Davey, Richard Keegan, Nicole Freene

**Affiliations:** 1 Health Research Institute, University of Canberra, Bruce, ACT, Australia; 2 Research Institute for Sports and Exercise (UCRISE), Faculty of Health, University of Canberra, Bruce, ACT, Australia; 3 Physiotherapy, Faculty of Health, University of Canberra, Bruce, ACT, Australia; Linneaus University, SWEDEN

## Abstract

**Background:**

Smartphone applications provide new opportunities for secondary prevention healthcare. This systematic review and meta-analysis aimed to determine if smartphone applications are effective at changing physical activity and sedentary behaviour in people with cardiovascular disease.

**Methods:**

Six electronic databases (Medline, CINAHL Plus, Cochrane Library, SCOPUS, Sports Discus and EMBASE) were searched from 2007 to October 2020. Cardiovascular disease secondary prevention physical activity or sedentary behaviour interventions were included where the primary element was a smartphone or tablet computer application (excluding SMS-only text-messaging). Study quality was assessed using validated tools appropriate for each study design. Random effects model was used and the pooled mean difference between post scores were calculated. Subgroup analyses were conducted to examine differences based on diagnosis, sample size, age, intervention duration, activity tracker use, target behaviour, and self-report versus device-measured outcome.

**Results:**

Nineteen studies with a total of 1,543 participants were included (coronary heart disease, n = 10; hypertension, n = 4; stroke, n = 3; heart failure, n = 1; peripheral artery disease, n = 1). Risk of bias was rated as high. Thirteen studies were included in the meta-analysis. Only two controlled studies reported on sedentary behaviour. Smartphone applications produced a significant increase of 40.35 minutes of moderate-to-vigorous intensity physical activity per week (7 studies; p = 0.04; 95% CI 1.03 to 79.67) and 2,390 steps per day (3 studies; p = 0.0007; 95% CI 1,006.9 to 3,791.2). Subgroup analyses found no difference when comparing diagnoses, sample size, activity tracker use, target behaviour and self-report versus device-measured outcome. Larger improvements in physical activity were noted in intervention durations of ≤3-months and participants ≥60yrs (95.35 mins.week^-1^; p = 0.05).

**Conclusions:**

Smartphone applications were effective in increasing physical activity in people with cardiovascular disease. Caution is warranted for the low-quality evidence, small sample and larger coronary heart disease representation. More rigorous research is needed to investigate the effect of smartphone applications across diagnoses and in sedentary behaviour.

## Introduction

Cardiovascular disease (CVD) is one of the leading causes of morbidity and mortality worldwide [1–3]. Increasing physical activity and decreasing sedentary behaviour are important secondary prevention strategies for people with CVD and contribute to reducing their risk of all-cause mortality [4]. Mobile health (mHealth) interventions continue to gain traction as alternative or adjunct therapies to influence physical activity for those with CVD.

With the increase of smartphones and applications (apps) across generations, mHealth provides the unique opportunity to influence and be integrated into patients’ daily life from ‘the pocket’. This is especially relevant during the COVID-19 pandemic where in-person delivery of secondary prevention strategies may not be possible [5]. Apps can provide direct line of contact to health professionals and with advances in technology can provide more sophisticated, automated and highly tailored interventions through means such as artificial intelligence (AI). Smartphone app interventions have the potential to be used as resource-efficient, secondary prevention tools for people with CVD, however, their efficacy for creating physical activity and sedentary behaviour change is unclear and their uptake across CVD diagnoses is unknown.

There have been multiple reviews of the use of mHealth for management of CVD and risk reduction over the last decade [6–8]. Pfaeffli et al. [6] completed a systematic review on mHealth behaviour change interventions for CVD self-management of which only included two studies reporting physical activity. No meta-analysis was completed. In 2017 Gandhi et al. [7] conducted a meta-analysis but studies included a majority of SMS text-messaging interventions. They found that people with CVD in the mHealth group showed a trend towards meeting exercise goals [7]. A meta-analysis was unable to be completed by Coorey et al. [8] on CVD and apps for self-management and risk factor control due to the large variation in study design and/or insufficient data within the studies. In these systematic reviews, physical activity is most frequently reported as steps per day (steps.day^-1^) and no meta-analysis has been conducted on minutes of moderate-to-vigorous intensity physical activity (MVPA) in participants with CVD. Global public health guidelines recommend adults with chronic conditions to do at least 150–300 minutes of moderate-intensity aerobic physical activity (MPA), or at least 75–150 minutes of MVPA per week, or an equivalent combination of both [9]. Furthermore, adults with chronic conditions should limit the amount of time spent in sedentary behaviour [9]. Patients with CVD should seek to accumulate 6,500 to 8,500 steps.day^-1^ in order to achieve a physical activity energy expenditure high enough to limit vascular disease progression [10, 11]. To date, the evidence for the use of smartphone apps to increase physical activity in people with CVD remains unclear.

Previous reviews have not focused exclusively on smartphone apps (excluding SMS-only text-messaging interventions). Compared to texting-based interventions, apps provide further opportunities for Bluetooth connected self-monitoring devices such as activity trackers, heart rate and blood pressure monitors. These provide the basis for individualised feedback and prompting and the opportunity for more interactive and engaging strategies for behaviour change. The use of apps for sedentary behaviour change is a newer area being explored in CVD research and hence there is no existing systematic review or meta-analysis examining the effect of smartphone apps on sedentary behaviour in this group. Such a review is required to help inform the further development of interventions to change physical activity and sedentary behaviour in those with CVD in ways that are scalable and of interest to this population.

The primary aim of this systematic review and meta-analysis was to determine if smartphone apps are effective at changing physical activity and sedentary behaviour for people with CVD. The secondary aims included: to determine the characteristics of participants with CVD who agree to engage with research involving smartphone apps (e.g. age, gender); to determine what factors appear to influence the uptake and engagement (e.g. length of intervention, use of activity trackers with the app, specifically targeting physical activity or sedentary behaviour); to understand the adherence rates to using smartphone apps by those with CVD; and to determine if there are differences in outcomes based on diagnostic group (e.g. coronary heart disease, stroke, peripheral artery disease).

## Methods

This systematic review is reported according to the Preferred Reporting Items for Systematic Reviews and Meta-Analyses statement (2020) (S1 Table) [12] and the Cochrane Handbook for Systematic Reviews of Interventions [13]. The protocol is registered with PROSPERO (CRD42020189046).

### Search strategy

An electronic database search was conducted using Medline, CINAHL Plus with Full Text (EBSCO), Cochrane Library, SCOPUS, Sports Discus and EMBASE. Search terms described the population group (CVD); intervention (smartphone applications); and the key outcome (physical activity and/or sedentary behaviour) (S1 File. Full search strategy). Peer-reviewed, English language, full-text studies of any type were included if published since the launch of the first app stores in 2007 to 31 October 2020. Reference lists of eligible studies and review articles were also screened. When suitable research protocol papers were found, a specific search was conducted in attempt to find the associated results paper. Results were imported into Covidence Systematic Review Software (Vertias Health Innovation, Melbourne, Australia, www.covidence.org) and duplicates were removed.

### Eligibility criteria

People aged >18yrs with diagnosed CVD, including coronary heart disease (CHD), heart failure, hypertension, cerebral vascular disease (stroke), peripheral artery disease, rheumatic heart disease, congenital heart disease, cardiomyopathies and cardiac arrhythmias [14], were included.

Secondary prevention interventions were included where the primary element was delivered through a smartphone or tablet computer app as either a standalone or as part of a multi-component intervention package (e.g. face-to-face cardiac rehabilitation or activity trackers such as smart watches). The aim of the app-based interventions may be to monitor or change physical activity or sedentary behaviour outcomes. Studies that had a broader health promotion goal related to CVD, such as blood pressure management, were included provided they reported on at least one post physical activity or sedentary behaviour outcome. Interventions which used only short messaging service (SMS), websites, video conferencing, telehealth or phone calls were excluded. The comparison group was either a true control group or active control group such as usual care.

Physical activity is any bodily movement that requires energy expenditure and includes activities such as exercise, recreation, active transport, physical work and household chores [15]. Sedentary behaviour can be defined as any sitting, reclining or lying posture behaviour characterized by energy expenditure of ≤1.5 metabolic equivalents [16]. Outcomes may be measured subjectively (e.g. self-report) or by a device such as an accelerometer.

Additional outcomes of interest to answer the secondary objectives included participant characteristics (e.g. age, gender, diagnoses) and the factors which influence uptake (including requirement to own a smartphone), engagement and adherence to such interventions as well as retention rates. Engagement was considered to be the extent (e.g. amount, frequency, duration, and depth) of usage, as well as the effort, involvement or interaction with using the smartphone app [17]. Adherence was considered to be the proportion of participants following the prescribed or intended activities as advised (e.g. recording daily steps) [18].

Published studies with a control group reporting on a post-intervention measure of physical activity or sedentary behaviour were eligible for inclusion in the meta-analysis (e.g. randomised controlled trials (RCTs) and quasi-experimental studies). Cohort studies were included in the narrative synthesis provided they reported a pre-post measure of physical activity or sedentary behaviour.

### Screening and selection process

**Fig 1:**
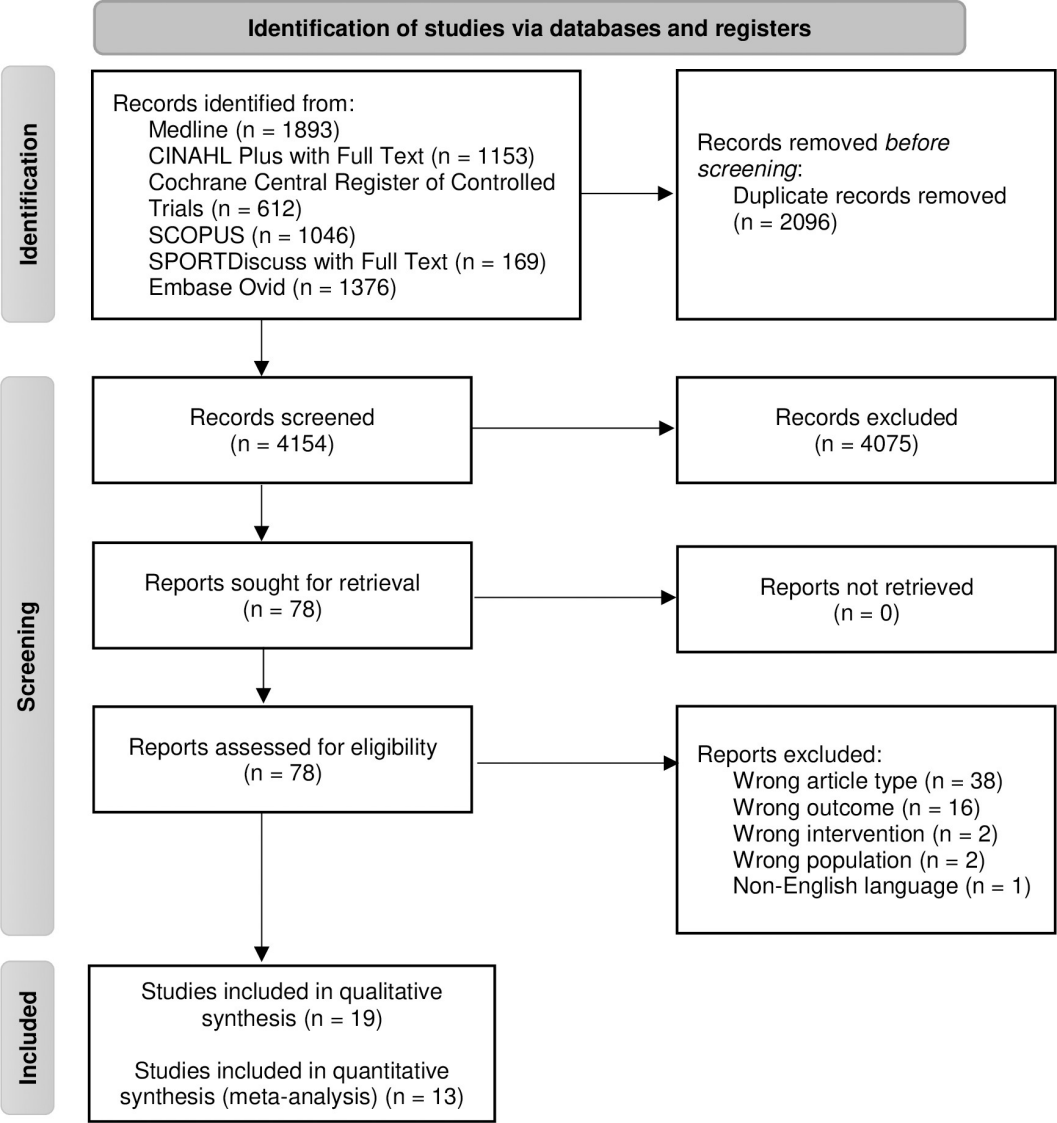
PRISMA flow diagram demonstrating the flow of studies through the review.

Two independent investigators screened titles and abstracts using a priori screening criteria and then the full-text articles were reviewed (KP and NF). Disagreements were discussed and resolved by consensus. A third investigator was not required to become involved. The study selection process is outlined in the PRISMA flow diagram (Fig 1).

### Data extraction

Information from the included studies was extracted by two independent investigators (KP and NF) to complete the Template for Intervention Description and Replication (TIDieR) Checklist [19]. A third reviewer was not required to resolve any discrepancies in data. Available information was sourced from published appendices, protocols, supporting studies and results papers. Additional information was extracted by one investigator (KP) into a Microsoft Excel spreadsheet and confirmed by a second (NF) including: study background; sample-related information at the point of randomisation; number and demographics of participants who were excluded or declined (if available); smartphone app-related information; comparator-related information; retention rates; adherence and engagement measures; and outcome-related information. Any discrepancies were resolved by discussion. Post-intervention means and standard deviation (SD) were recorded where possible. For one study [20], the following equation was used to calculate the post-intervention mean and SD:

$${Mean}_{Change}={Mean}_{endpoint}-{Mean}_{baseline}$$

$${SD}_{Change}=\sqrt{{\left({SD}_{baseline}\right)}^{2}+{\left({SD}_{endpoint}\right)}^{2}-(2\times r\times {SD}_{baseline}\times {SD}_{endpoint})}$$ (1)

This study [20] did not report the correlation coefficient (*r*) in order to calculate the SD_*endpoint*_ for post-intervention exercise minutes per week, therefore the correlation was conservatively set at 0.5 [21, 22]. In addition, a sensitivity analysis was conducted using a correlation of 0.2 and 0.8 for comparison. This did not significantly change the outcome. Medians were assumed as means for the purpose of the meta-analysis [23] for one study [24]. Studies that reported only moderate-intensity physical intensity (MPA) were included with MVPA as the contribution of vigorous-intensity physical activity in these populations is likely to be small [24, 25]. MVPA minutes per day were converted to minutes per week (MVPA mins.week^-1^) by multiplying by 7 [26]. Weekly minutes of exercise were assumed to be MVPA mins.week^-1^ [20, 24, 27]. In two studies [28, 29], a Fitbit Charge was used to collect activity data. The parameters for what this equate to is not openly available and therefore the authors of both studies equated Fitbit “light” activity to activities of daily living, “fairly active” to moderate-low intensity physical activity and “very active” to moderate-high intensity physical activity. Corresponding authors were emailed where additional information was required.

### Risk of bias in individual studies

Studies were assessed for bias by two independent investigators (KP and NF). The internal validity of RCTs was appraised using the Revised Cochrane risk-of-bias (RoB-2) [30]. For quasi-experimental and non-randomised control trials (non-RCTs), the ROBIN-I tool was used [31]. For studies without control groups, the Quality Assessment Tool for Before-After (Pre-Post) Studies With No Control Group was used [32].

### Strategy for data synthesis

A meta-analysis was conducted on physical activity and sedentary behaviour outcomes for studies that had a control group. Outcomes were included in the meta-analysis if data was available from three or more studies.

Review Manager (RevMan 5.4.1) computer software was used for the meta-analyses using generic inverse variance with random effects model to calculate the pooled mean difference with 95% confidence intervals [33]. The *I*^2^ statistic describes the percentage of total variation across studies that is attributed to heterogeneity rather than chance [34]. Heterogeneity was interpreted in accordance with the Cochrane guidelines: 0–40% low and might not be important, 30–60% may represent moderate, 50–90% may represent substantial and 75–100% considerable heterogeneity [35]. The associated *p* values of <0.1 indicate heterogeneity is unlikely to be due to chance alone [34]. A funnel plot was not considered appropriate to check for publication bias due to less than ten studies being included in each meta-analysis [13].

Subgroup analyses were completed to compare participants (CHD vs other CVD; age <60yrs versus ≥60yrs; ≤50 participants versus >50 participants); and intervention (activity tracker with app versus app only; length ≤3-months versus >3-months; targeting physical activity versus other health behaviours). Sensitivity analyses were completed to examine the effect of methods used (MPA versus MVPA; self-report versus device-measured).

### Non-statistical analysis

All studies were included in the narrative synthesis of study design, participant and intervention characteristics, measurement type of physical activity and sedentary behaviour and potential to change physical activity or sedentary behaviour. Interventions were classified as being either adaptive or static in their delivery mode. Adaptive interventions were context-aware, individualised, tailored and changing throughout the duration of the delivery dependent on how the participant was responding [36]. Examples of this include investigators changing step goals based on the participant achieving the goal [28, 29, 37] or more sophisticated methods such as using algorithms, machine learning or AI to tailor the intervention based off the input data [25, 38–42]. When an intervention set parameters, daily reminders or a prescription (e.g. steps or exercise minutes per week) for the participant at the beginning and did not alter this based on performance, it was considered static.

Studies were classified into three categories based on their potential to change physical activity or sedentary behaviour using the same method to Gardner et al. [43]. Interventions were ‘Very promising’ if there was a statistically significant increase in physical activity or reduction in sedentary behaviour between the intervention group and the comparator arm. This excluded all single arm studies. “Quite promising” interventions were when there was either significant changes in physical activity or sedentary behaviour (for cohort studies) or when at least one indicator was improved but did not reach significance compared to the comparator arm (for multi-arm studies). The “Non-promising” classification was used when there were no physical activity or sedentary behaviour changes [43]. Narrative comparisons of studies in each of these categories were conducted to explore possible explanatory factors.

## Results

### Study selection

The database search retrieved 4,154 articles (Fig 1). Full-text screening was conducted on 78 articles. Additional information was requested from authors of 8 articles, with 4 authors replying and able to supply the required information [20, 27, 44, 45]. The other 4 articles were excluded as not meeting the inclusion criteria due to lack of reported outcome measures. Nineteen studies were included in the qualitative synthesis (Fig 1). There were 13 studies with control groups however there was large variation in the measures used for physical activity and sedentary behaviour and hence meta-analyses were only able to be completed for MVPA mins.week^-1^ [20, 24–26, 28, 29, 38] and steps.day^-1^ [28, 29, 37].

### Study characteristics

Of the 19 studies included in the review, 10 were RCTs, 3 were non-RCTs, and 6 were cohort design. All studies were published within the last 5yrs. Study characteristics are summarized in Table 1. Majority of the studies were either feasibility or pilot trials (68.4%, 13/19) [25–29, 37, 39–42, 44–46]. Most included studies used a usual care control group [20, 25–28, 37, 45, 47–49] or a lower functioning version of an app [24, 38]. Half the studies had ≤50 participants (52.6%, 10/19). Duration of intervention ranged from 3-weeks to 12-months with the majority running for six or more months [24, 38, 40, 44, 47–49]. Only one study specifically targeted sedentary behaviour [39], all others either explicitly targeted physical activity or a range of health-related behaviours for CVD secondary prevention including physical activity (Table 1). Seven studies used an activity tracker such as a smartwatch or external pedometer as part of the intervention [28, 29, 39–42, 44]. Six studies provided their participants with a smartphone to participate in the study [29, 37, 40–42, 47]. Adverse events were reported in two studies however were deemed as not being directly related to the app intervention [25, 37].

**Table 1:** Summary of included studies.

Study	Participants	Intervention	Outcome measures	Key findings
Duscha, 2018a [28] RCT, USA	n = 25 (Exp 16, Con 9)	12-weeks	Device-measured (Fitbit): steps.day^-1^, low PA mins.day^-1^, low PA mins.week^-1^, moderate-low PA mins.day^-1^, moderate-low PA mins.week^-1^, moderate-high PA mins.day^-1^, moderate-high PA mins.week^-1^, moderate PA mins.week^-1^, total active mins.day^-1^, total active mins.week^-1^	Exp = NS ↑ steps.day^-1^, moderate-high PA mins.day^-1^ and mins.week^-1^
		Target behaviour = PA		
	Age (yr) = Exp 59 (SD 8.1), Con 66.5 (SD 7.2)			
		Exp = Vida Health app and commercial Fitbit app–Fitbit Charge tracking + health coaching		
				Con = NS ↓ steps.day^-1^, low PA mins.day^-1^ and mins.week^-1^, total active mins.day^-1^ and mins.week^-1^. Significant ↓ moderate-low PA mins.week^-1^ (117±78 vs 50±53; p<0.05), moderate-high PA mins.week^-1^ (111±87 vs 65±64; p<0.05).
	F (%) = 24%			
	Dx = MI ± revascularization (PCI or CABG), valve repair, heart failure or stable angina	Intensity = Weekly prompts		
		Purpose = Sustain PA patterns and monitor daily steps		
	Setting = Outpatient phase-II CR	Con = Usual care–no specific lifestyle recommendations from study personnel		
				Between group difference in moderate-high PA mins.week^-1^ (Exp 21±103 vs Con -46±36; p<0.05)
			Follow up = Baseline, 12-weeks	
Duscha, 2018b [29] RCT, USA	n = 20 (Exp 10, Con 10)	12-weeks	Device-measured (Fitbit): steps.day^-1^, low PA mins.day^-1^, low PA mins.week^-1^, moderate-low PA mins.day^-1^, moderate-low PA mins.week^-1^, moderate-high PA mins.day^-1^, moderate-high PA mins.week^-1^, moderate PA mins.week^-1^, total active mins.day^-1^, total active mins.week^-1^	Exp = NS ↑ steps.day^-1^, moderate-low PA mins.day^-1^ and mins.week^-1^, moderate-high PA mins.day^-1^ and mins.week^-1^, total active mins.day^-1^ and mins.week^-1^
		Target behaviour = PA		
	Age (yr) = 69 (SD 8.4)			
		Exp = Commercial Fitbit app + Fitbit Charge activity tracker + web-based PAD patient information book		
	F (%) = 15.8%			
	Dx = PAD with intermittent claudication who were sedentary			
				Con = NS ↑ steps.day^-1^ and NS ↓ in all other PA outcomes.
		Intensity = Phone call if did not meet the prescribed steps for 2 consecutive weeks + weekly email with PAD tip of the week		
	Setting = Outpatient cardiology clinic			Between group difference in moderate-high PA mins.day^-1^ (Exp 10±17 vs Con -2±4; p<0.05) and mins.week^-1^ (Exp 69±122 vs Con -14±31; p<0.05)
		Purpose = Monitor daily steps		
			Baseline, 12-weeks	
		Con = Hardcopy of PAD information book and physician guidance–no specific lifestyle recommendations from study personnel		
Freene, 2020 [39] Pre-Post, Australia	n = 20	6-weeks	Device-measured (accelerometer–Actigraph ActiSleep): MVPA mins.day^-1^, LPA mins.day^-1^, SB mins.day^-1^, % SB.day^-1^, duration of SB bounts.day^-1^(mins), number of SB bouts.day^-1^, number of SB breaks.day^-1^, vector magnitude counts.day^-1^, steps.day^-1^	Effect size for ↓ in SB mins.day^-1^ was medium (Cohen d = 0.54) and small for % SB.day^-1^ (Cohen d = 0.25) at 16 weeks.
	Age (yr) = 54 (SD 13)	Target behaviour = SB		
		Exp = Vire app and ToDo-CR behaviour change program + Fitbit Flex + GPS data		
	F (%) = 15%			
				NS ↓ in duration of SB bounts.day^-1^(mins), number of SB bouts.day^-1^, and number of SB breaks.day^-1^ at 16-weeks.
	Dx = CR participants including stable CHD, CABG, PCI, MI			
		Intensity = Automatic messages generated from a bank by the system using machine learning. 14–19 push notifications during the 6-week intervention		
				NS ↑ in MVPA mins.day^-1^, LPA mins.day^-1^, vector magnitude counts.day^-1^, and steps.day^-1^ at 6-weeks and 16-weeks.
	Setting = Outpatient phase-II CR			
		Purpose = Self-monitoring activity levels and improve behavioural flexibility to change SB		
			Baseline, 6-weeks, 16-weeks	
		Con = N/A		
Grau-Pellicer, 2020 [26] RCT, Spain	n = 41 (Exp 24, Con 17)	8-weeks	Self-report: community ambulation mins.day^-1^, sitting time hours.day^-1^	Exp = ↑ community ambulation mins.day^-1^ (56.85 ± 52.81; P≤0.05; Cohen’s d = 2.58) and ↓ sitting time hours.day^-1^ (2.96 ± 2.07; P≤0.05; Cohen’s d = 1.22).
		Target behaviour = PA (community ambulation) + SB		
	Age (yr) = Exp 62.96 (SD 11.87), Con 68.53 (SD 11.53)			
			Baseline, 3-months	
		Exp = Fitlab Training app and Fitlab Test app including GPS and accelerometer monitoring + WhatsApp		Con = NS ↑ community ambulation mins.day^-1^ and NS ↓ sitting time hours.day^-1^.
	F (%) = 48.7%			
	Dx = Chronic stroke			
		Intensity = Unclear		
	Setting = Community dwelling	Purpose = Supervise adherence to PA guidelines by monitoring walking distance and speed		
		Con = Usual care–conventional 3-month face-to-face rehabilitation program		
Johnston, 2016 [24] Multicenter RCT, Sweden	n = 166 (Exp 86, Con 80)	6-months	Self-report (unspecified questionnaire): MPA mins.week^-1^, number of PA sessions.week^-1^, % exercise >150 min.week^-1^	Both groups NS ↑ MPA mins.week^-1^, number of PA sessions.week^-1^, % exercise >150 min.week^-1^
		Target behaviour = Medication adherence + achievement of secondary prevention targets		
	Age (yr) = Exp 56.8 (SD 8), Con 58.4 (SD 8.6)			
	F (%) = 19%	Exp = SUPPORT app = e-diary for drug adherence + interactive patient support tool for information modules and recording data		
	Dx = MI		Baseline, 6-months	
	Setting = Traditional secondary prevention care including CR			
		Intensity = Every second day during the first 2 weeks, then 3 messages per week		
		Purpose = Monitor drug adherence and lifestyle behaviours including exercise, weight management and smoking		
		Con = Simplified smartphone drug adherence e-diary = report daily use of tricagrelor (medication) without feedback or education modules. Generic SMS if missed a dose.		
Kim, 2016 [47] Sub-study of RCT, USA	n = 95 (Exp 52, Con 43)	6-months	Self-report (Godin Leisure-time Exercise Questionnaire): weekly leisure activity score	Both groups NS ↑ weekly leisure activity score
		Target behaviour = Hypertension control- frequency of the use of alcohol, smoking, and exercise		
	Age (yr) = 57.6 (SD 8.6)			
	F (%) = 68%			
			Baseline, 6-months	
	Dx = Hypertension	Exp = HealthyCircles app = Wireless self-monitoring program + disease management + Withings Blood Pressure Monitor		
	Setting = Community dwelling accessing health facilities			
		Intensity = Encouraged to use 3x per week and take 2 measurements per day. If the participant did not meet the frequency for 2 weeks, a reminder email was sent		
		Purpose = Wireless self-monitoring of BP and education		
		Con = Usual care–HealthComp disease management program		
		Both = HealthComp disease management program with HealthComp relaying medical education		
Lunde, 2020 [48] Multicenter RCT, Norway	n = 113 (Exp 57, Con 56)	12-months	Self-report (interview): number of 30min moderate-to-vigorous intensity exercise sessions per week. Baseline, 12-months	Exp = ↑ number of 30min moderate-to-vigorous intensity exercise sessions per week within group change from baseline to 12-moths (1.4±1.5, p<0.001)
		Target behaviour = PA		
	Age (yr) = 59 (SD 8.7)			
		Exp = Vett app = activity monitoring and feedback + goal setting + communicate with supervisor. Intensity = Motivational feedback 1-3x per week based on individual preference. Purpose = Set goals and reminders related to health behaviours. Con = Usual care–general advice according to a heart-friendly lifestyle and follow-up by their general practitioner		
	F (%) = 22.1%			
	Dx = Heart disease including 73.4% CAD, 16.8% valve surgery and 9.8% other heart diseases			
				Con = ↑ number of 30min moderate-to-vigorous intensity exercise sessions per week within group change from baseline to 12-moths (0.6±1.1, p<0.001)
	Setting = Varying CR programs across inpatient and outpatients			
				Between group difference favoured Exp number of 30min moderate-to-vigorous intensity exercise sessions per week (0.9, 95% CI 0.4–1.4; p<0.001).
Lv, 2017 [40] Pre-Post, USA	n = 149	6-months	Self-report (Stanford Exercise Behaviour Scale): mins.week^-1^ aerobic exercise, mins.week^-1^ stretching or strengthening	↑ mins.week^-1^ aerobic exercise (178.6±132.4 vs 206.4±126.2,P = 0.03)
	Age (yr) = 62.2 (SD 9.5)	Target behaviour = PA		
				No change mins.week^-1^ stretching or strengthening (P = 0.91).
	F (%) = 51%	Exp = EMPOWER-H app + Numera app + Web-based dashboard + Nurse Care Manager + wireless BP cuff, pedometer		
	Dx = Hypertension			
			Baseline, 6-months	
	Setting = Ambulatory healthcare system			
		Intensity = Instructed to measure and upload BP 2x day for at least 3 days per week and upload daily step count		
		Purpose = Support timely patient-provider interaction and personalized feedback for chronic disease management		
		Con = N/A		
Nabutovsky, 2020 [44] Pre-Post, Israel	n = 22	6-months	Device-measured (smartwatch with matching smartphone Polar application—Polar Inc, M430; Kempele, Finland): steps.day^-1^, aerobic exercise mins.week^-1^, frequency of aerobic exercise.week^-1^, frequency of resistance exercise.week^-1^, attainment of 150min.week^-1^ of aerobic exercise. Baseline, 6-months	↓ frequency of aerobic exercise.week^-1^ (P = 0.03) and ↑ frequency of resistance exercise.week^-1^ (P = 0.02). NS ↓ steps.day^-1^ and aerobic exercise mins.week^-1^. No reported change in attainment of 150min.week^-1^ of aerobic exercise at follow-up.
	Age (yr) = 52.7 (SD 5.5)	Target behaviour = PA. Exp = Tele-CR with Datos Health app + care-team dashboard + smartwatch with Polar application. Intensity = Tele-health specialist consultation weekly via the messaging system or phone call. Exercise program updated every 5-weeks. Purpose = Remote monitoring, communication and management for secondary prevention. Con = N/A		
	F (%) = 22.7%			
	Dx = CAD			
	Setting = Outpatient cardiac prevention and rehabilitation centre			
Paul, 2016 [37] Non-RCT, Scotland	n = 24 (Exp 16, Con 8)	6-weeks	Device-measured (accelerometer–Actigraph ActivPAL): steps.day^-1^, sedentary time hours.day^-1^, upright time hours.day^-1^, walking time hours.day^-1^	Exp = NS ↑ steps.day^-1^, upright time hours.day^-1^, and walking time hours.day^-1^. NS ↓ sedentary time hours.day^-1^.
		Target behaviour = PA		
	Age (yr) = 56 (SD 10)			
		Exp = STARFISH mobile phone app which users’ PA is visualized by fish swimming in a tank in virtual groups of 4		
				Con = NS ↓ steps.day^-1^, walking time hours.day^-1^ and sedentary time hours.day^-1^. NS ↑ upright time hours.day^-1^.
	F (%) = 52%			
	Dx = Stroke			
			Baseline, 6-weeks	
				For group-time interaction, steps.day^-1^ ↑ by 39.3% (4158 to 5791 steps.day^-1^) in Exp and ↓ by 20.2% (3694 to 2947 steps.day^-1^) in Con (P = 0.005; and a large effect, partial η2 = 0.314). Similar group-time interaction occurred for walking time hours.day^-1^ with ↑ in Exp walking time hours.day^-1^ by 20mins and ↓ in Con by 14mins (P = 0.002; and a large effect, partial η2 = 0.381).
		Intensity = Weekly increase in step goal		
	Setting = Stroke support groups			
		Purpose = Behavioural change intervention to encourage the user to become more physically active		
		Con = Usual care–no active rehabilitation, only appointments with health care professionals as required		
Persell, 2020 [38] RCT, USA	n = 333 (Exp 166, Con 167)	6-months	Self-report (unspecified questionnaire): MVPA mins.week^-1^. Baseline, 6-months	Exp = NS ↑ MVPA mins.week^-1^. Con = NS ↑ MVPA mins.week^-1^. NS between-group differences.
	Age (yr) = 58.9 (SD 12.8)	Target behaviour = Hypertension control–PA, diet, medication adherence, BP measurement, sleep, stress management. Exp = Hypertension Personal Control Program (HPCP) = Artificial Intelligence (AI) technology + HPCP coaching app + blood pressure monitor. Intensity = Prompts daily BP measurement in the first week, then weekly prompts thereafter. Unspecified frequency of reminders for medication, PA, weight and diet. Purpose = Promote home-monitoring of BP and behaviour changes associated with hypertension self-management. Con = Omron app—Blood pressure tracking app + home blood pressure monitor, plus routine care as prescribed by their regular clinicians		
	F (%) = 61.3%			
	Dx = Uncontrolled hypertension			
	Setting = Community dwelling receiving care from outpatient clinics			

Requena, 2019 [45] Non-RCT, Spain	n = 159 (Exp 107, Con 52)	3-4-weeks	Self-report (unclear): achieving >30 mins.day^-1^	No baseline measures completed. Between-group difference at 90 days for achieving >30 mins.day^-1^ was NS.
	Age (yr) = 58.4 (SD 11.4)			
	F (%) = 44%	Target behaviour = PA + medication adherence. Exp = Farmalarm app for secondary prevention of stroke through vascular risk factor control = register obs + GPS + education + communicate with medical staff. Intensity = Medication alarm reminder at each time it is due. Contact with stroke team could be scheduled as requested by the participant. Purpose = Increase stroke awareness by medication alerts, chat communication with medical staff, didactic video files and exercise monitoring. Con = Usual care–stroke unit 90 day follow-up visit		
	Dx = Stroke			
	Setting = Community dwelling following discharge home			
			90 days	
Salvi, 2018 [25] Multicenter RCT, Spain, Germany and UK	n = 118 (Exp 55, Con 63)	21-weeks	Self-report (custom questionnaire): MPA mins.week^-1^	Statistical test not performed due to large drop-outs and poor data collection.
		Target behaviour = PA		
	Age (yr) = 58 (SD 10)		Baseline, 6-months	
		Exp = HeartCycle GEx system available on smartphone and tablet = Mobile Station + Patient Station + Professional Station		
				Exp = ↑ MPA mins.week^-1^
	F (%) = 11%			Con = ↓ MPA mins.week^-1^
	Dx = CR graduates with CAD			
		Intensity = Automatic feedback generated by the system with on average 163 messages sent to each participant		
	Setting = Community dwelling following completion of phase-II CR			
		Purpose = Motivate patients to adhere to their rehabilitation program through exercise monitoring, guidance, motivational feedback and educational content		
		Con = Usual care–Phase 3 standard rehabilitation according to the national procedures of each of the 3 countries. Also asked to report on daily PA on a paper diary		
Sengupta, 2020 [41] Pre-Post, USA	n = 10	12-weeks	Self-report (International Physical Activity Questionnaire-Short Form): days of MPA, MPA mins.day^-1^, sitting mins.day^-1^, days walked at least 10mins.day^-1^. Baseline, 12-weeks	NS ↑ in days of MPA, MPA mins.day^-1^, sitting mins.day^-1^, and days walked at least 10mins.day^-1^
	Age (yr) = 64.4 (SD 6.3)	Target behaviour = PA + diet. Exp = HerBeat app for smartphone and smartwatch = collect data on daily PA, HR, eating episodes, mood + personalised messages within the app. Intensity = Weekly encouragement to engage with the app. Purpose = To help women with behavioural self-management through goal-setting, tracking progress and educational videos. Con = N/A		
	F (%) = 100%			
	Dx = ACS or coronary revascularization in the last 10 years			
	Setting = Outpatient cardiology clinic			
Song, 2020 [49] RCT, China	n = 106 (Exp 53, Con 53)	6-months	Self-report (unclear): exercise habits according to meeting the American College of Sports Medicine 10^th^ edition of exercise testing and prescription	Exp = ↑ proportion with exercise habits
		Target behaviour = PA		Con = ↑ proportion with exercise habits
	Age (yr) = Exp 54.17 (SD 8.76), Con 54.83 (SD 9.13)			
				Between-group difference in exercise habit favouring Exp (93.8% vs 77.1%; P = 0.02).
		Exp = Telemonitoring software called MEMRS-CRS and WeChat app = HR monitoring (with belt) during exercise + personalised feedback. Intensity = Weekly feedback. Purpose = Provide remote CR through telemonitoring including exercise (following FITT principle) and vital signs. Con = Usual care–routine discharge education and outpatient follow-up with advice to exercise regularly. Both = Routine discharge education and outpatient follow-up which included advice to exercise regularly		
	F (%) = 13.5%			
	Dx = Stable CHD		Baseline, 6-months	
	Setting = Community dwelling			
Weerahandi, 2020 [46] Pre-Post, USA	n = 17	120-days	Device-measured (wireless pedometer): steps.day^-1^	NS ↑ steps.day^-1^
	Age (yr) = 59 (SD 6)	Target behaviour = Hypertension control–PA, diet, weight, BP measurement. Exp = DASH Mobile app + track diet, blood pressure, weight, PA + human coach and coach-facing web-based portal. Intensity = Coach interaction once per week via instant message, SMS or email. Purpose = Track diet, BP, weight, daily PA and coaching for improved hypertension management. Con = N/A		
	F (%) = 60%			
	Dx = Hypertension			
			Baseline (days 1–7), follow-up (days 46–120)	
	Setting = Community dwelling			
Werhahn, 2019 [42] Pre-Post, Germany	n = 10	2-months	Device-measured (smartphone—iPhone 6SE, Apple Inc., Cupertino, CA, USA, iOS Versions 10.2.1–11.2.1 + smartwatch—Apple Watch 1st Gen., Apple Inc., watchOS Versions 3.1.1–4.2.2): steps.day^-1^	↑ steps.day^-1^ (3612 ± 3311 to 7069 ± 5006; p<0.0001)
	Age (yr) = 46.3 (SD 7.8)	Target behaviour = PA		
	F (%) = 40%	Exp = Cardio patient monitoring platform (CPMP) for smartphone and Apple smartwatch + Physician’s tablet. Intensity = Optional reminders for medication. Encouraged daily input of vital signs and activity monitoring. Purpose = Remote monitoring through collecting HF symptoms, medication adherence, PA data and vital signs. Con = N/A		
	Dx = Newly diagnosed HF with reduced ejection fraction			
	Setting = Community following discharge from hospital			
			Baseline, 1-month, 2-months	
Widmer, 2015 [27] Non-RCT, USA	n = 44 (Exp 25, Con 19)	3-months	Self-report (Health behaviour questionnaire): exercise mins.week^-1^	Exp = ↑ exercise mins.week^-1^ (148.1±78.5, p<0.0001)
	Age (yr) = Exp 60.2 (SD 12.1), Con 70.4 (SD 9.9)	Target behaviour = CVD risk factors–PA, weight, BP measurement, diet, laboratory values. Exp = Personal Health Assistant = integrated and personalised interface that tracks, logs, educates and forms actionable tasks. Intensity = Daily reminders for height, weight, BP, lab values, PA and diet. Occasional email reminders if the participant had not logged in recently. Purpose = Tracks, logs, educates and forms actionable tasks for the user to improve health. Con = Usual care–Mayo Clinic phase II CR program for 36 sessions. Both = Standard Mayo Clinic CR program for 36 sessions (approximately 3 months)		
	F (%) = 18.2%			
	Dx = PCI for ACS			Con = ↑ exercise mins.week^-1^ (117.3±61.6, p<0.0001)
	Setting = Outpatient phase-II Mayo Clinic CR			
			Baseline, 3-months	
				NS between-group difference
Widmer, 2017 [20] RCT, USA	n = 71 (Exp 37, Con 34)	3-months	Self-report (unspecified questionnaire): exercise mins.week^-1^	Exp = NS ↑ exercise mins.week^-1^
	Age (yr) = Exp 62.5 (SD 10.7), Con 63.6 (SD 10.9)	Target behaviour = PA + diet		Con = NS ↑ exercise mins.week^-1^
		Exp = Personal Health Assistant (digital health intervention) and web-based application = patients to report dietary and exercise habits throughout CR + educational information. Intensity = Asked participants to log in 3–4 times weekly. Purpose = Tracks, logs, educates and forms actionable tasks for the user to improve health. Con = Usual care–Mayo Clinic phase II CR program for 36 sessions. Both = Standard Mayo Clinic CR program for 36 sessions (approximately 3 months)		
	F (%) = 18.3%			
	Dx = PCI for ACS		Baseline, 3-months	NS between-group difference—however the intervention group increased by more (179 vs 139mins)
	Setting = Outpatient phase-II Mayo Clinic CR			

Note abbreviations: ACS, Acute coronary syndrome; App, Application; CR, Cardiac rehabilitation; CVD, Cardiovascular disease; Con, Control group; CABG, Coronary artery bypass graft surgery; CAD, Coronary artery disease; CHD, Coronary heart disease; Dx, Diagnosis; Exp, Experimental group; F, Female; HF, Heart failure; LPA, Light-intensity physical activity; MPA, Moderate-intensity physical activity; MVPA, Moderate-to-vigorous intensity physical activity; MI, Myocardial infarction; NS, Non-significant; PCI, Percutaneous coronary intervention; PAD, Peripheral artery disease; PA, Physical activity; RCT, Randomised control trial; SB, Sedentary behaviour; SD, Standard deviation.

### Quality assessment

**Fig 2:**
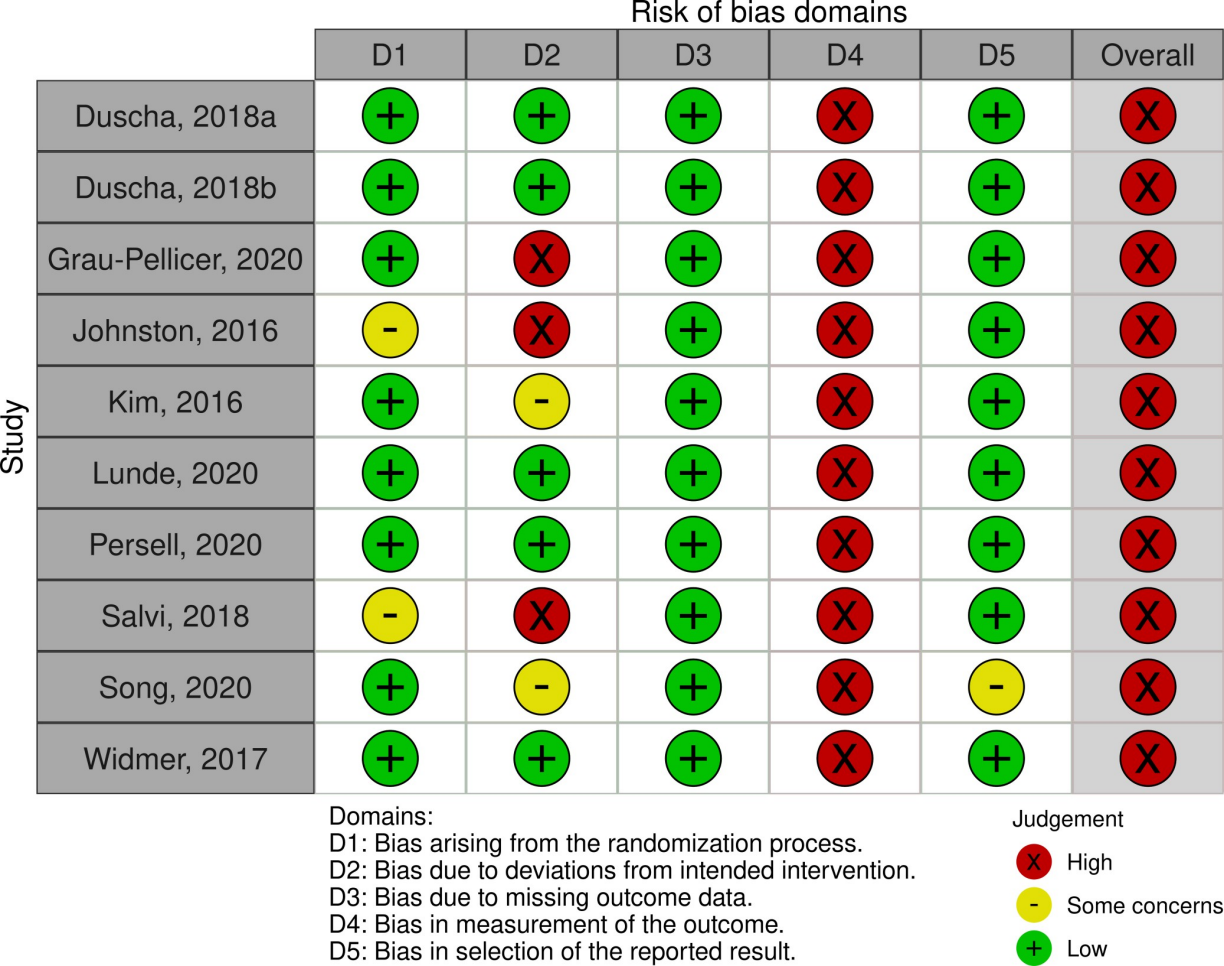
Randomised controlled trials appraised using the Revised Cochrane risk-of-bias (RoB-2).

**Fig 3:**
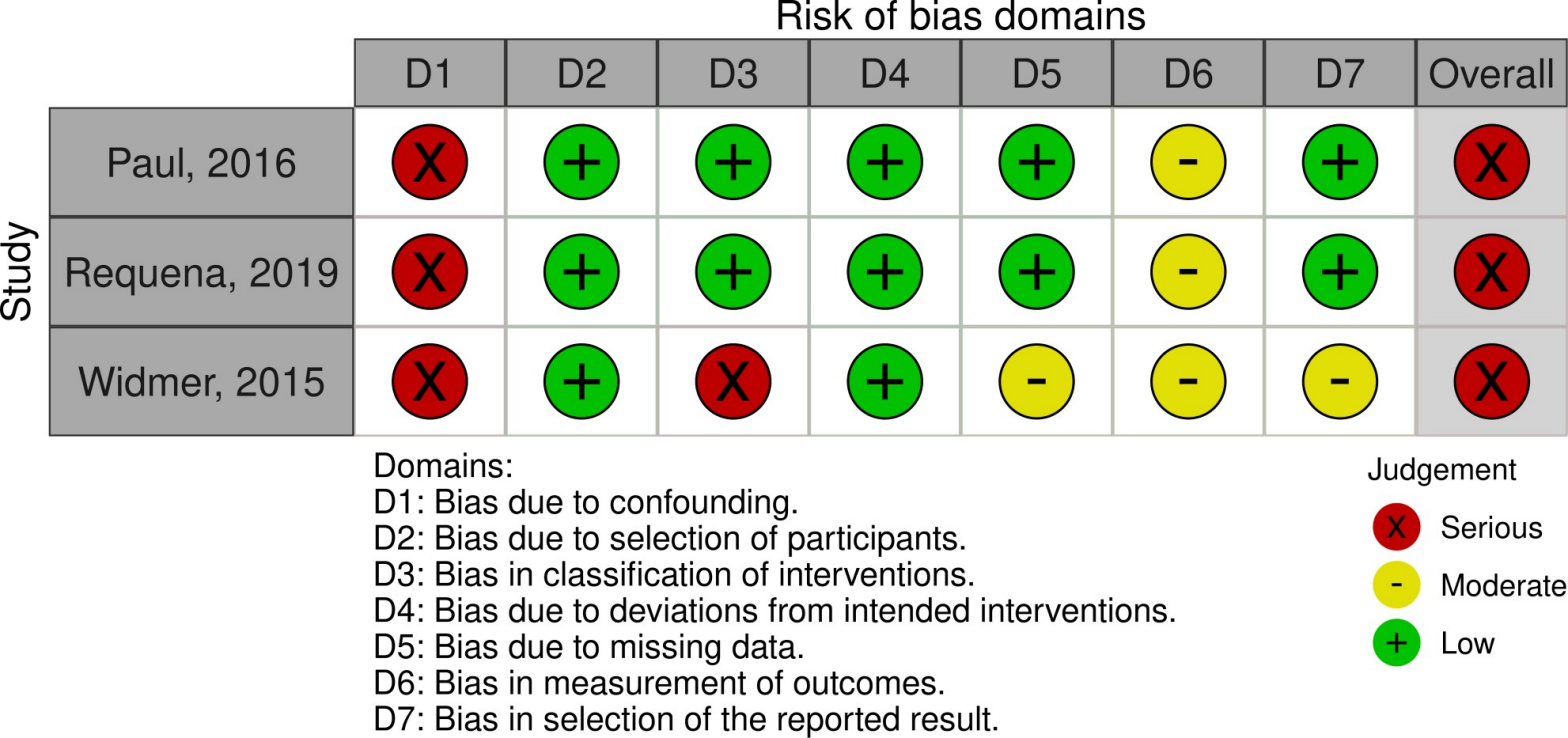
Quasi-experimental and non-randomised studies, appraised using the ROBIN-I tool.

**Fig 4:**
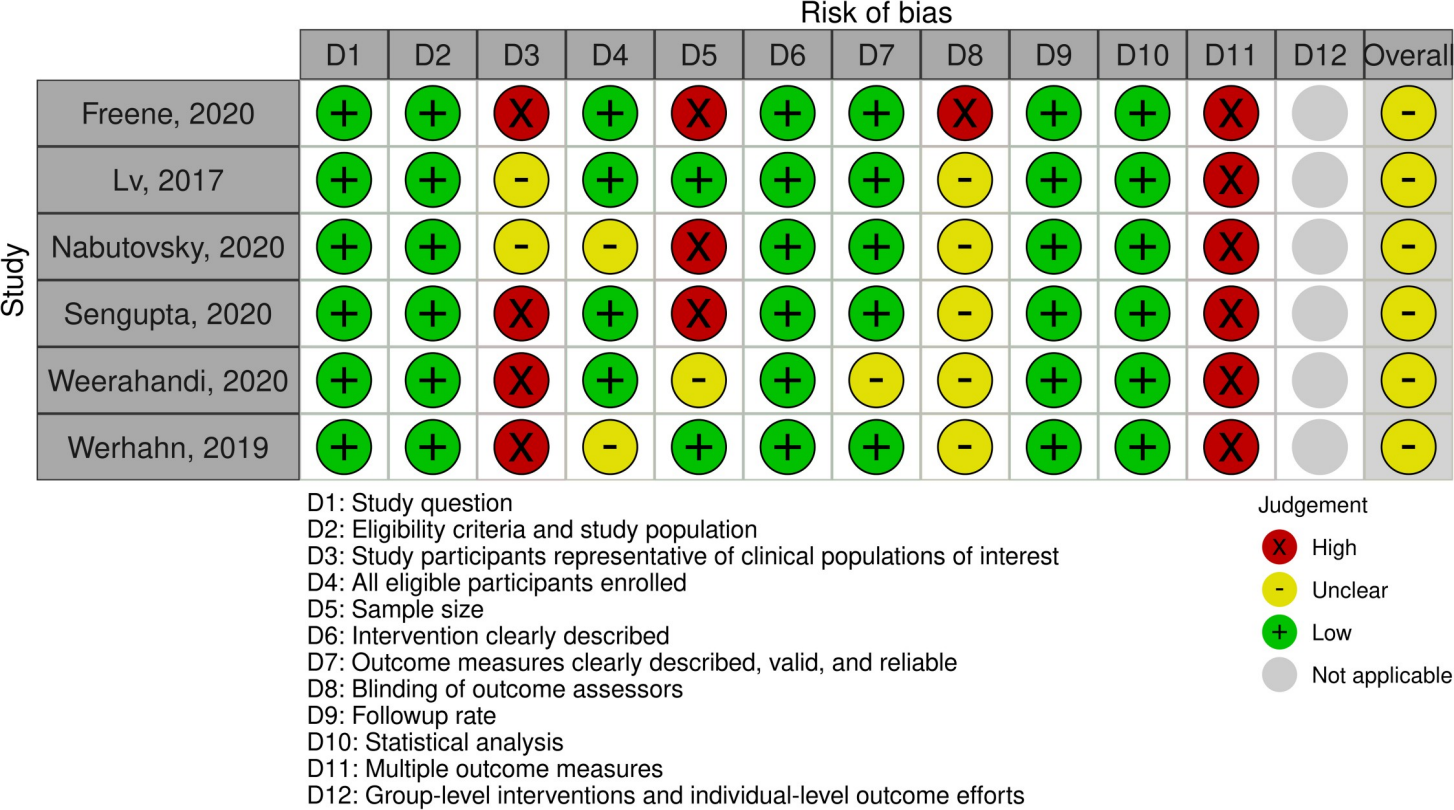
Studies without control groups, appraised using the Quality Assessment Tool for Before-After (Pre-Post) Studies with No Control Group.

All ten RCTs [20, 24–26, 28, 29, 38, 47–49] and three non-RCTs [27, 37, 45] had high and serious risk of bias respectively (Figs 2 and 3). The cohort studies were all considered fair [39–42, 44, 46] (Fig 4). Due to the nature of the intervention and the outcome, blinding of participants, outcome assessors and people assisting in the delivery of the intervention, was minimally used. Self-report physical activity and sedentary behaviour were used by the majority of studies [20, 24–27, 38, 40, 41, 45, 47–49]. Pedometers or smartwatches were used to measure activity in some studies [28, 29, 42, 44, 46], however, there is potential bias as the participants could visually see their outcomes. This resulted in high risk of bias in measurement of the outcome. Accelerometry was seldomly used to measure physical activity and sedentary behaviour [37, 39] despite this being a more valid and reliable method [50].

### Participant characteristics

The majority of studies included participants with CHD (52.6%, 10/19) [20, 24, 25, 27, 28, 39, 41, 44, 48, 49]. Other studies included those with diagnosed hypertension [38, 40, 46, 47], stroke [26, 37, 45], HF [42], and PAD [29]. The number of participants involved in 19 studies was 1,543 with a mean age of 59.7yrs (range 46.3yrs [42] to 69yrs [29]; SD 5.5). The proportion of male participants, compared to female, was greater in 14 studies and one study included only women [41]. Participants were required to own and use a smartphone in 9 studies (47.4%) [24, 28, 38, 39, 44–46, 48, 49]. Three studies preferred participants to own a smartphone but it was not considered an exclusion criteria (15.8%) [20, 26, 27]. In 6 studies (31.6%), participants were provided with a smartphone to participate [29, 37, 40–42, 47] and one study was unclear [25]. One study reported that those without a smartphone were significantly older than those with a smartphone (mean difference 20 ± 5yrs, p < 0.001) [39].

### Change in physical activity

There was a wide range of outcomes used to report physical activity (Table 1). The majority of studies used self-report measures including questionnaires and interviews (63.2%, 12/19). The main measure used was MVPA mins.week^-1^ (n = 12) [20, 24–29, 38–41, 44], then steps.day^-1^ (n = 7) [28, 29, 37, 39, 42, 44, 46] and exercise frequency (n = 6) [24, 41, 44, 45, 48, 49]. Common characteristics of studies (S2 Table) which reported a significant between-group increase in physical activity and hence ‘very promising’ were: RCT study design (71.4%, 5/7), included ≤50 participants (71.4%, 5/7/), ≤3-months in duration (71.4%, 5/7), involved participants with an average age ≥60yrs (57.1%, 4/7), targeted physical activity only (71.4%, 5/7), and used an adaptive mode of delivery (85.7%, 6/7). Non-promising interventions (n = 9), as defined by not causing change in physical activity outcomes [43], had the following key features: 55.6% included >50 participants (n = 5/9), 66.7% targeted multiple health behaviours (n = 6/9), 88.9% used an adaptive delivery mode (n = 8/9), and 66.7% involved participants with a mean age of 55-60yrs (n = 6/9).

#### Moderate-to-vigorous intensity physical activity minutes per week

**Fig 5:**
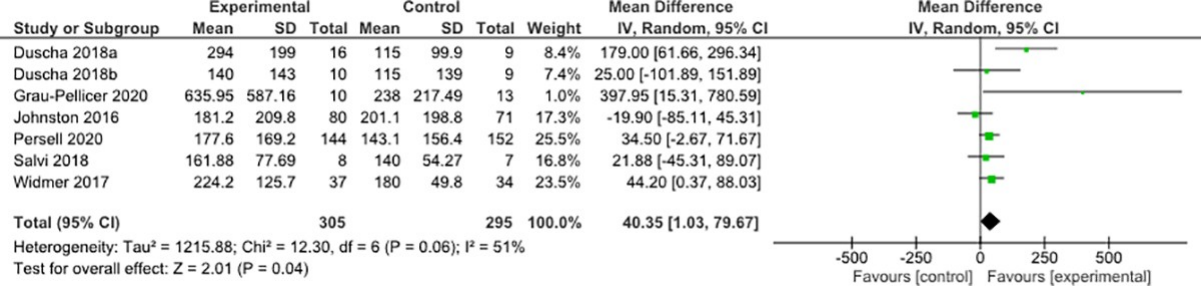
Mean differences and 95% confidence intervals between groups for MVPA mins.week^-1^.

Seven RCTs measured MVPA mins.week^-1^ including 305 participants in the experimental group and 295 in the control [20, 24–26, 28, 29, 38]. MVPA mins.week^-1^ was significantly different between groups with a pooled mean difference of 40.35 mins.week^-1^ (95% CI 1.03 to 79.67; p = 0.04; Fig 5) in favour of the experimental group. There was moderate heterogeneity (*I*^2^ = 51%; *χ*^2^ = 12.30; p = 0.06). Sensitivity analysis found no difference when comparing MVPA versus MPA and self-report versus device measured. Five of the included seven studies in this meta-analysis used self-report measures of MVPA. Subgroup analyses found no difference when comparing CHD versus all other CVD and ≤50 participants versus >50 participants. When just including studies of 3-months or less [20, 26, 28, 29], there was a statistically significant increase in MVPA mins.week^-1^ of 95.35 minutes (95% CI -0.16 to 190.86; p = 0.05; *I*^2^ = 61%; *χ*^2^ = 7.74; p = 0.05) (Fig a in S2 File). These same four studies were also the only studies with a mean participant age ≥60yrs. When including studies with a mean participant age <60yrs [24, 25, 38], MVPA mins.week^-1^ was no longer significant (Fig b in S2 File). The MVPA mins.week^-1^ significantly increased by 28.3 minutes with low heterogeneity (95% CI 4.06 to 52.55; p = 0.02; *I*^2^ = 0%; *χ*^2^ = 2.75; p = 0.43) when including interventions that only used an app [20, 24, 25, 38] (Fig c in S2 File). However, studies which used an activity tracker with the app [26, 28, 29] non-significantly increased by 141.83 mins.week^-1^ (95% CI -14.19 to 297.85; p = 0.07; *I*^2^ = 61%; χ^2^ = 5.15; p = 0.08) (Fig d in S2 File). Studies which specifically targeted physical activity [25, 26, 28, 29] non-significantly increased MVPA by 93.40 mins.week^-1^ (95% CI -14.94 to 201.75; p = 0.09; *I*^2^ = 65%; χ^2^ = 8.48; p = 0.04) (Fig e in S2 File).

#### Steps per day

**Fig 6:**
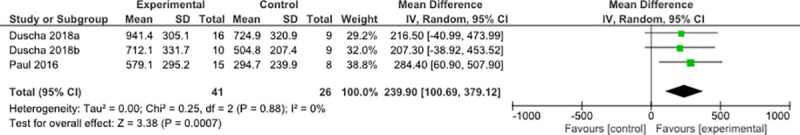
Mean differences and 95% confidence intervals between groups for steps.day^-1^. Note: For graphical purposes, the steps data was divided by 10.

Two RCTs and one non-RCT measured steps.day^-1^ including 41 participants in the experimental group and 26 in the control [28, 29, 37]. All three studies were device-measured, specifically targeted physical activity, were ≤3-months duration and included <50 participants. Steps.day^-1^ was significantly different between groups with a pooled mean difference of 2,390 steps.day^-1^, (95% CI 1,006.9 to 3,791.2; p = 0.0007; Fig 6) in favour of the experimental group with low heterogeneity (*I*^2^ = 0%; *χ*^2^ = 0.25; p = 0.88). Subgroup analyses were not completed due to the low number of studies and participants.

### Change in sedentary behaviour

Only four studies measured sedentary behaviour: one RCT [26], one non-RCT [37], and two cohort studies [39, 41] (S3 Table). All four studies were ≤3-months in duration and included ≤50 participants. For 50% (2/4) of the studies, participant mean age was ≥60yrs [26, 41]. Accelerometers [37, 39] were used in two studies and self-report in the other two [26, 41]. Only one study had a statistically significant between group change in sedentary behaviour, and therefore was ‘very-promising’ [26]. The two cohort studies had no change in sedentary behaviour [39, 41]. Only one study specifically targeted sedentary behaviour [39]. One study was static in its delivery and was the only study with a significant change in sedentary behaviour [26]. The two studies with a change in sedentary behaviour both were designed for participants who had a stroke [26, 37].

### Smartphone app intervention uptake, engagement, adherence and drop-out

#### Uptake and support

Those who agreed to participate in the included studies were mostly men (61.8%, 953/1,543) mean age 59.7yrs. Participants were excluded from participating based on not having a smartphone [28, 38, 39, 48] or due to having problems with the technology [24, 48] (S4 Table). A small to large number of participants declined to participate for unspecified reasons [20, 28, 38–40, 48, 49]. Further analyses were not possible for the characteristics of participants who were not included in the studies due to limited information available. Five (5/19, 26.3%) studies provided their participants with the smartphone and apps already downloaded and showed them how to use the apps [37, 40–42, 47]. Eleven (11/19, 57.9%) studies worked with participants to help them download the apps to their own phone and get orientated to using the apps [20, 24, 26–29, 38, 44, 46, 48, 49]. Two (2/19, 10.5%) studies provided participants with instructions and left them to download the apps on their own, providing support when needed [39, 45] and one study was unclear on the level of support for participants [25].

#### Engagement and adherence

Four studies did not report on engagement or adherence to the app intervention [28, 37, 45, 49] (S4 Table). The definitions of engagement and adherence varied and were not clearly stated in majority of studies. Adherence to the app intervention was mentioned in 12 studies (63.2%) [20, 24–26, 29, 39, 40, 42, 44, 46–48] and ranged from 20% [29] to 84.8% [42]. Similarly, engagement with the app intervention was mentioned in 8 studies [25, 27, 29, 38, 40, 41, 46, 47] with a vast range of measures, including number of logged activities to having conversations with the app, making it difficult to draw conclusions. Due to not all studies reporting on adherence or engagement, comparisons against physical activity sedentary behaviour change could not be completed. Adherence and/or engagement was noted to decline over the intervention period [29, 40, 47].

Six studies reported that technical problems may have impacted on participation. The main cause of low rate of use or drop-outs related to the app were feelings of it being too challenging, forgetting to wear monitoring devices, problems with internet connection or synching devices, and safety algorithms preventing the completion of exercise sessions [25, 26, 44, 46, 47, 49]. One study also reported on the need to increase the level of supports for downloading and setting up the app for both study participants and clinical staff due to low confidence with technology [39].

#### Drop-outs

The drop-out rates varied from 0% [20, 41, 45, 46] to 87% [25], with no clear difference between groups (S4 Table). One study [39] completed a follow-up measure beyond completion of the intervention which reported increased drop-out rate from completion of the intervention (6-weeks, 5%) to final follow-up (16-weeks, 40%). A small proportion of participants dropped out due to the technology being too complex, having to wear a tracking device continuously or feelings of demotivation due to problems with the system [25, 44, 49].

## Discussion

This meta-analysis suggests that smartphone apps for people with CVD are effective at increasing physical activity. Both MVPA mins.week^-1^ and steps.day^-1^ significantly increased compared to the controls. Subgroup analyses suggest further increases in physical activity outcomes when interventions were short-term (≤ 3-months). It is unclear if smartphone apps are effective in reducing sedentary behaviour for people with CVD due to the low number of published studies. Men aged 60yrs appeared to have the highest uptake of included studies. This is typical given the majority of participants had diagnosed CHD. There were varying levels of adherence and engagement and problems with the apps or technology, but these minimally impacted on participation. These results should be interpreted with caution due to low number of studies, small sample sizes (half the studies having ≤ 50 participants), high risk of bias due to lack of blinding, limited representation of each CVD diagnostic group and majority of studies relying on self-report outcomes. Research into apps has only recently become important and demonstrates the infancy of smartphone apps for physical activity and sedentary behaviour change in people with CVD.

The overall change in steps reported in the meta-analysis would equate to an approximate increase of 20-minutes of walking per day [51]. This is comparable with other similar sized and larger meta-analyses measuring steps.day^-1^ by people with various chronic diseases including CVD using wearable activity trackers [52–54]. It remains uncertain if the mean increase in steps observed in this review is clinically meaningful. There are no published minimal clinically important differences for CVD or the included diagnoses of CHD, hypertension, stroke, heart failure and peripheral artery disease. In the similar patient population of chronic obstructive pulmonary disease, a minimal clinically important difference of 350 to 1,100 steps.day^-1^ is reported which also resulted in a reduction in hospital admissions [55, 56]. The app interventions in this review appear to meet this minimal clinically important difference however caution is required as there were only three studies included in the meta-analysis for steps.day^-1^.

The authors could find no other meta-analyses of MVPA in CVD with smartphone app interventions. The improvement in MVPA mins.week^-1^ found in this review was higher than that found in a meta-analysis of 12 studies by Kirk et al. who investigated wearable activity tracker interventions in a cardiometabolic disease population [53]. The higher increase in MVPA could perhaps be explained by the additional behaviour change techniques possible with more interactive interventions delivered through apps compared to simply tracking activity. The use of the activity tracker in combination with an app is a behaviour change technique which may help participants by self-monitoring their behaviour, goal setting, action planning, and drawing attention to discrepancies between current behaviour and goal [57]. In the current study, there was no additional benefit for using an activity tracker with the app. Though this was a small sample of studies, there has been no previous reviews comparing CVD apps with or without use of an activity tracker. The larger meta-analysis and meta-regression in general population by Laranjo et al. [58] found no statistically significant difference between physical activity outcomes when the intervention used an activity tracker or just a smartphone app. It appears, with or without an activity tracker, smartphone apps can increase physical activity.

The increases in physical activity were larger in short interventions with participants over 60yrs. Though these studies were small (≤ 50 participants) and few in number, it is consistent with a previous meta-analysis showing larger improvements in MVPA mins.week^-1^ in studies less than 16-weeks [53]. This may be due to motivation or commitment to behaviour change being greater in these first few months compared to longer interventions. Interventions of longer duration had larger sample sizes and younger cohorts. This may demonstrate the lack of efficacy of apps for longer term use. In addition, given these studies generally targeted multiple health behaviours associated with CVD, this may also be the cause for lack of physical activity change.

Interestingly, targeting physical activity did not statistically change MVPA. In comparison, a review of smartphone apps for increasing physical activity in a combination of healthy adults and those with chronic disease found significant increases in steps.day^-1^ when physical activity was targeted in isolation [59]. When activity trackers were used in combination with the smartphone app, sedentary behaviour did not change. This is consistent with evidence showing that interventions for physical activity, such as activity trackers, do not necessarily change sedentary behaviour [43, 60, 61]. Evidence for the use of activity trackers, independently of other intervention components, on inducing changes in sedentary behaviour is somewhat limited. However, previous reviews outside of CVD populations [62, 63] report that often activity trackers are used as part of dual aim interventions to reduce sedentary behaviour by shifting to light and then to more moderate-vigorous physical activities. This is an area requiring further exploration in CVD populations. Future interventions may benefit from explicitly targeting physical activity or sedentary behaviour.

It does not appear that outcomes differed based on diagnosis of CHD compared to any other CVD diagnosis. This has not been reported in other meta-analyses however, there was limited representation of each CVD diagnoses. The level of support for uptake in the intervention has not been previously summarized in other reviews either. The time and level of assistance should be considered when assessing the feasibility of running such interventions in real world settings where clinical staff time is sparse for additional workload tasks. Furthermore, it was not noted if additional support was required for older participants. A post hoc analysis of app usage stratified by age showed that participants at or above the median age of 61yrs, had more conversations with the app than the participants below the median age in one study [38]. In contrast, another study found no significant associations between usage frequency and age [27]. Therefore, age does not appear to be a limiting factor for engagement in such interventions.

Adherence and engagement were inconsistently reported making it difficult to draw any conclusions. Kirk et al. [53] found only 42.9% of the included studies reported some form of intervention adherence compared to this study (63.2%). However, their reported adherence rates were substantially higher compared to this review (63.3% to 100% vs 20% to 84.8%) and found that intervention adherence reduced with time. Future research would benefit by setting clear definitions of adherence and engagement, tracking how these change over time and whether this is associated with physical activity or sedentary behaviour change. This would help define the ideal frequency of engagement with the app and duration of the intervention in order to create the greatest changes in behaviour.

This review incorporated a rigorous search strategy following a pre-defined protocol registration. Additional research questions regarding what behaviour change techniques were used by the included studies and their impact on increasing physical activity and decreasing sedentary behaviour were beyond the scope of this review. An additional review will be completed to address these unanswered questions. Subgroup analyses were completed to evaluate possible explanatory factors such as intervention length, age and effectiveness with or without use of activity trackers. However, the available evidence was subject to high risk of bias in all RCTs and non-RCTs and should be interpreted within this context. Most outcomes were self-report and hence subject to bias. The range of physical activity measures used resulted in meta-analyses only being able to be completed on two outcomes and one of which was very small. There was also moderate heterogeneity in the studies when interpreting the MVPA mins.week^-1^ and the subgroup analyses included small numbers of studies. The primary research question relating to sedentary behaviour was unable to be answered with low numbers of published papers. The purpose of the included studies must also be taken into consideration. Many were feasibility studies or pilot RCTs and hence were not necessarily designed to detect a change in physical activity or sedentary behaviour. Furthermore, some of the included studies would not apply to a real world setting as it is unlikely facilities would supply smartphones. This limits the generalizability of the results and points to the need for larger scale RCTs. Lastly, there was limited information available to address the secondary aims of this review and as such not all planned subgroup analyses were able to be completed. This information is required in order to successfully translate research into practice in a meaningful way.

## Conclusion

Smartphone apps were effective in increasing physical activity for those with CVD and the effects were unclear for sedentary behaviour. The results indicate a potential benefit of smartphone apps in CVD patients, although caution is warranted due to the low-quality evidence to date and most being in coronary heart disease. Interventions appear to be most effective when short in duration (≤ 3-months) and in older adults. These findings demonstrate the infancy of smartphone applications in this population, especially for sedentary behaviour change. Larger scale research is needed to investigate the effect of smartphone applications across CVD diagnoses to be able to determine ideal length and components of successful interventions.

## Supporting information

S1 Table PRISMA checklist. (DOCX)

S2 Table Potential to change physical activity. (DOCX)

S3 Table Potential to change sedentary behaviour. (DOCX)

S4 Table Uptake, engagement, adherence and drop-outs. (DOCX)

S1 File Full search strategy. (DOCX)

S2 File Moderate to vigorous intensity physical activity minutes per week subgroup analysis results. (DOCX)
