# Supplementary material for: Smartphone applications for physical activity and sedentary behaviour change in people with cardiovascular disease: A systematic review and meta-analysis
Source: PLoS One. 2021 Oct 11;16(10):e0258460. doi: 10.1371/journal.pone.0258460 (PMC8504773; doi:10.1371/journal.pone.0258460)
Supplement: S1 Table — (DOCX) [file pone.0258460.s001.docx]

| **Section and Topic** | **Item #** | **Checklist item** | **Location where item is reported** |
| --- | --- | --- | --- |
| **TITLE** | | |  |
| Title | 1 | Identify the report as a systematic review. | p1, line 1 |
| **ABSTRACT** | | |  |
| Abstract | 2 | See the PRISMA 2020 for Abstracts checklist. | p2, line 27 |
| **INTRODUCTION** | | |  |
| Rationale | 3 | Describe the rationale for the review in the context of existing knowledge. | p3, line 32-76 |
| Objectives | 4 | Provide an explicit statement of the objective(s) or question(s) the review addresses. | p5, line 77-85 |
| **METHODS** | | |  |
| Eligibility criteria | 5 | Specify the inclusion and exclusion criteria for the review and how studies were grouped for the syntheses. | p6, line 104-134 |
| Information sources | 6 | Specify all databases, registers, websites, organisations, reference lists and other sources searched or consulted to identify studies. Specify the date when each source was last searched or consulted. | P5, line 93-102 |
| Search strategy | 7 | Present the full search strategies for all databases, registers and websites, including any filters and limits used. | p6, line 93-102 & S1 File |
| Selection process | 8 | Specify the methods used to decide whether a study met the inclusion criteria of the review, including how many reviewers screened each record and each report retrieved, whether they worked independently, and if applicable, details of automation tools used in the process. | P7, line 136-139 & Fig 1 |
| Data collection process | 9 | Specify the methods used to collect data from reports, including how many reviewers collected data from each report, whether they worked independently, any processes for obtaining or confirming data from study investigators, and if applicable, details of automation tools used in the process. | p8, line 144-153 |
| Data items | 10a | List and define all outcomes for which data were sought. Specify whether all results that were compatible with each outcome domain in each study were sought (e.g. for all measures, time points, analyses), and if not, the methods used to decide which results to collect. | P6, line 118-134 & p8, line 144-154 |
|  | 10b | List and define all other variables for which data were sought (e.g. participant and intervention characteristics, funding sources). Describe any assumptions made about any missing or unclear information. | p8, line 144-154 |
| Study risk of bias assessment | 11 | Specify the methods used to assess risk of bias in the included studies, including details of the tool(s) used, how many reviewers assessed each study and whether they worked independently, and if applicable, details of automation tools used in the process. | p9, line 172-176 |
| Effect measures | 12 | Specify for each outcome the effect measure(s) (e.g. risk ratio, mean difference) used in the synthesis or presentation of results. | P9, line 181-187 |
| Synthesis methods | 13a | Describe the processes used to decide which studies were eligible for each synthesis (e.g. tabulating the study intervention characteristics and comparing against the planned groups for each synthesis (item #5)). | p9, line 178-180 |
|  | 13b | Describe any methods required to prepare the data for presentation or synthesis, such as handling of missing summary statistics, or data conversions. | P8, line 155-170 |
|  | 13c | Describe any methods used to tabulate or visually display results of individual studies and syntheses. | N/A |
|  | 13d | Describe any methods used to synthesize results and provide a rationale for the choice(s). If meta-analysis was performed, describe the model(s), method(s) to identify the presence and extent of statistical heterogeneity, and software package(s) used. | P9, line 178-192 & p10, line 194-215 |
|  | 13e | Describe any methods used to explore possible causes of heterogeneity among study results (e.g. subgroup analysis, meta-regression). | Not completed |
|  | 13f | Describe any sensitivity analyses conducted to assess robustness of the synthesized results. | P9, line 188-192 |
| Reporting bias assessment | 14 | Describe any methods used to assess risk of bias due to missing results in a synthesis (arising from reporting biases). | Not completed |
| Certainty assessment | 15 | Describe any methods used to assess certainty (or confidence) in the body of evidence for an outcome. | P9, 181-187 |
| **RESULTS** | | |  |
| Study selection | 16a | Describe the results of the search and selection process, from the number of records identified in the search to the number of studies included in the review, ideally using a flow diagram. | p11, line 219-226 & Fig 1 |
|  | 16b | Cite studies that might appear to meet the inclusion criteria, but which were excluded, and explain why they were excluded. | Fig 1 & p11, line 219-226 |
| Study characteristics | 17 | Cite each included study and present its characteristics. | p11, line 228-241 & Table1 |
| Risk of bias in studies | 18 | Present assessments of risk of bias for each included study. | p22, line 287-297 & Figs 2-4 |
| Results of individual studies | 19 | For all outcomes, present, for each study: (a) summary statistics for each group (where appropriate) and (b) an effect estimate and its precision (e.g. confidence/credible interval), ideally using structured tables or plots. | Table 1 & Figs 5-6 |
| Results of syntheses | 20a | For each synthesis, briefly summarise the characteristics and risk of bias among contributing studies. | p23, line 251-260 & p25, line 298-304 |
|  | 20b | Present results of all statistical syntheses conducted. If meta-analysis was done, present for each the summary estimate and its precision (e.g. confidence/credible interval) and measures of statistical heterogeneity. If comparing groups, describe the direction of the effect. | p25, line 298-316 & p26, line 334-343 & Figs 5-6 & S2 File |
|  | 20c | Present results of all investigations of possible causes of heterogeneity among study results. | p25, line 301-312 |
|  | 20d | Present results of all sensitivity analyses conducted to assess the robustness of the synthesized results. | p25, line 302-3316 & p26, line 327-328 & S2 File |
| Reporting biases | 21 | Present assessments of risk of bias due to missing results (arising from reporting biases) for each synthesis assessed. | Not completed |
| Certainty of evidence | 22 | Present assessments of certainty (or confidence) in the body of evidence for each outcome assessed. | p25, line 300-302 & p26, line 325-327 |
| **DISCUSSION** | | |  |
| Discussion | 23a | Provide a general interpretation of the results in the context of other evidence. | p28, line 385-464 |
|  | 23b | Discuss any limitations of the evidence included in the review. | p32, line 465-482 |
|  | 23c | Discuss any limitations of the review processes used. | p32, line 465-482 |
|  | 23d | Discuss implications of the results for practice, policy, and future research. | p31, line 442-443, p31, line 460-464 & p32, line 485-493 |
| **OTHER INFORMATION** | | |  |
| Registration and protocol | 24a | Provide registration information for the review, including register name and registration number, or state that the review was not registered. | p5, line 90 |
|  | 24b | Indicate where the review protocol can be accessed, or state that a protocol was not prepared. | p5, line 90 |
|  | 24c | Describe and explain any amendments to information provided at registration or in the protocol. | P26, line 372-373, p26, line 334-335, p28, line 365-367 |
| Support | 25 | Describe sources of financial or non-financial support for the review, and the role of the funders or sponsors in the review. | N/A |
| Competing interests | 26 | Declare any competing interests of review authors. | none |
| Availability of data, code and other materials | 27 | Report which of the following are publicly available and where they can be found: template data collection forms; data extracted from included studies; data used for all analyses; analytic code; any other materials used in the review. | On request |

*From:*  Page MJ, McKenzie JE, Bossuyt PM, Boutron I, Hoffmann TC, Mulrow CD, et al. The PRISMA 2020 statement: an updated guideline for reporting systematic reviews. BMJ 2021;372:n71. doi: 10.1136/bmj.n71

For more information, visit: <http://www.prisma-statement.org/>
