# Supplementary material for: Smartphone applications for physical activity and sedentary behaviour change in people with cardiovascular disease: A systematic review and meta-analysis
Source: PLoS One. 2021 Oct 11;16(10):e0258460. doi: 10.1371/journal.pone.0258460 (PMC8504773; doi:10.1371/journal.pone.0258460)
Supplement: S2 Table — (DOCX) [file pone.0258460.s002.docx]

**S2 Table. Potential to change physical activity**

| **Characteristics** | **Potential to change physical activity** | | | |
| --- | --- | --- | --- | --- |
|  | **Very promising**  **(n = 7) (%)**  Duscha, 2018a; Duscha, 2018b; Grau-Pellicer, 2020; Lunde, 2020; Paul, 2016; Song, 2020; Widmer, 2015 | **Quite promising**  **(n = 3) (%)**  Lv, 2017; Werhahn, 2019; Widmer, 2017 | **Non-promising**  **(n = 9) (%)**  Freene, 2020; Johnston, 2016; Kim, 2016; Nabutovsky, 2020; Persell, 2020; Requena, 2019; Salvi, 2018; Sengupta, 2020; Weerahandi, 2020 | **Total**  **(n = 19) (%)** |
| **Study design**  RCT  Non-RCT  Single cohort (pre-post) | 5 (71.4%)  2 (28.6%)  N/A | 1 (33.3%)  0  2 (66.7%) | 4 (44.4%)  1 (11.1%)  4 (44.4%) | 10 (52.6%)  3 (15.8%)  6 (31.6%) |
| **Sample size (total)**  ≤ 50  51-100  101-150  151-200  >200 | 5 (71.4%)  0  2 (28.6%)  0  0 | 1 (33.3%)  1 (33.3%)  1 (33.3%)  0  0 | 4 (44.4%)  1 (11.1%)  1 (11.1%)  2 (22.2%)  1 (11.1%) | 10 (52.6%)  2 (10.5%)  4 (21.1%)  2 (10.5%)  1 (5.3%) |
| **Participant mean age**  < 50 years  50-55 years  55-60 years  > 60 years | 0  1 (14.3%)  2 (28.6%)  4 (57.1%) | 1 (33.3%)  0  0  2 (66.7%) | 0  2 (22.2%)  6 (66.7%)  1 (11.1%) | 1 (5.3%)  3 (15.8%)  8 (42.1%)  7 (36.8%) |
| **CVD diagnosis**  CHD (i.e. MI, PCI or CABG, stable angina)  HF  Hypertension  Stroke  PAD | 4 (57.1%)  0  0  2 (28.6%)  1 (14.3%) | 1 (33.3%)  1 (33.3%)  1 (33.3%)  0  0 | 5 (55.6%)  0  3 (33.3%)  1 (11.1%)  0 | 10 (52.6%)  1 (5.3%)  4 (21.1%)  3 (15.8%)  1 (5.3%) |
| **Duration of intervention**  ≤ 3-months  3-5-months  ≥ 6-months | 5 (71.4%)  0  2 (28.6%) | 2 (66.7%)  0  1 (33.3%) | 3 (33.3%)  2 (22.2%)  4 (44.4%) | 10 (52.6%)  2 (10.5%)  7 (36.8%) |
| **Mode of measurement**  Self-report  Device-measured (i.e. smartwatch, pedometer, accelerometer) | 4 (57.1%)  3 (42.8%) | 2 (66.7%)  1 (33.3%) | 6 (66.7%)  3 (33.3%) | 12 (63.2%)  7 (36.8%) |
| **Outcome**  Steps  Minutes of LPA  Minutes of MPA  Minutes of MPVA  Total minutes of PA  Exercise frequency  Walking time  Upright time  VM magnitude counts  Weekly leisure activity score | 3 (42.8%)  0  2 (28.6%)  3 (42.9%)  2 (28.6%)  2 (28.6%)  1 (14.3%)  1 (14.3%)  0  0 | 1 (33.3%)  0  1 (33.3%)  1 (33.3%)  0  0  0  0  0  0 | 3 (33.3%)  1 (11.1%)  2 (22.2%)  3 (33.3%)  0  4 (44.4%)  0  0  1 (11.1%)  1 (11.1%) | 7 (36.8%)  1 (5.3%)  5 (26.3%)  7 (36.8%)  2 (10.5%)  6 (31.6%)  1 (5.3%)  1 (5.3%)  1 (5.3%)  1 (5.3%) |
| **Health behaviour targeted**  PA only  PA and SB  SB  Other health behaviours including PA | 5 (71.4%)  1 (14.3%)  0  1 (14.3%) | 2 (66.7%)  0  0  1 (33.3%) | 2 (22.2%)  0  1 (11.1%)  6 (66.7%) | 9 (47.4%)  1 (5.3%)  1 (5.3%)  8 (42.1%) |
| **Activity tracker used in the intervention** | 2 (28.6%) | 2 (66.7%) | 3 (33.3%) | 7 (36.8%) |
| **Delivery mode**  Static  Adaptive (changing across the intervention based on participation) | 1 (14.3%)  6 (85.7%) | 0  3 (100%) | 1 (11.1%)  8 (88.9%) | 2 (10.5%)  17 (89.5%) |

Note: *Very promising* = where significant increases in at least one physical activity indicator between the intervention group and the comparator arm. This excludes all single arm studies. *Quite promising* = where there were either significant changes in at least one physical activity outcome (for cohort studies) within the intervention group or when at least one physical activity outcome was improved but did not reach significance compared to one comparator arm (for multi-arm studies). *Non-promising* = where there were neither physical activity changes within the intervention arm nor differences relative to at least one comparator arm.
