# Supplementary material for: Smartphone applications for physical activity and sedentary behaviour change in people with cardiovascular disease: A systematic review and meta-analysis
Source: PLoS One. 2021 Oct 11;16(10):e0258460. doi: 10.1371/journal.pone.0258460 (PMC8504773; doi:10.1371/journal.pone.0258460)
Supplement: S3 Table — (DOCX) [file pone.0258460.s003.docx]

**S3 Table. Potential to change sedentary behaviour**

| **Characteristics** | **Potential to change sedentary behaviour** | | | |
| --- | --- | --- | --- | --- |
|  | **Very promising**  **(n = 1) (%)**  Grau-Pellicer, 2020 | **Quite promising**  **(n = 1) (%)**  Paul, 2016 | **Non-promising**  **(n = 2) (%)**  Freene, 2020; Sengupta, 2020 | **Total**  **(n = 4) (%)** |
| **Study design**  RCT  Non-RCT  Single cohort (pre-post) | 1 (100%)  0  N/A | 0  1 (100%)  0 | 0  0  2 (100%) | 1 (25%)  1 (25%)  2 (50%) |
| **Sample size (total)**  ≤ 50  51-100  101-150  151-200  >200 | 1 (100%)  0  0  0  0 | 1 (100%)  0  0  0  0 | 2 (100%)  0  0  0  0 | 4 (100%)  0  0  0  0 |
| **Participant mean age**  < 50 years  50-55 years  55-60 years  > 60 years | 0  0  0  1 (100%) | 0  0  1 (100%)  0 | 0  1 (50%)  0  1 (50%) | 0  1 (25%)  1 (25%)  2 (50%) |
| **CVD diagnosis**  CHD (i.e. MI, PCI or CABG, stable angina)  HF  Hypertension  Stroke  PAD | 0  0  0  1 (100%)  0 | 0  0  0  1 (100%)  0 | 2 (100%)  0  0  0  0 | 2 (50%  0  0  2 (50%)  0 |
| **Duration of intervention**  ≤ 3-months  3-5-months  ≥ 6-months | 1 (100%)  0  0 | 1 (100%)  0  0 | 2 (100%)  0  0 | 4 (100%)  0  0 |
| **Mode of measurement**  Self-report  Device-measured (i.e. smartwatch, pedometer, accelerometer) | 1 (100%)  0 | 0  1 (100%) | 1 (50%)  1 (50%) | 2 (50%)  2 (50%) |
| **Outcome**  Sitting time  Duration of SB bouts  Number of SB bouts  Number of SB breaks | 1 (100%)  0  0  0 | 1 (100%)  0  0  0 | 2 (100%)  1 (50%)  1 (50%)  1 (50%) | 4 (100%)  1 (25%)  1 (25%)  1 (25%) |
| **Health behaviour targeted**  SB only  PA only  SB and PA  Other health behaviour including PA | 0  0  1  0 | 0  1 (100%)  0  0 | 1 (50%)  0  0  1 (50%) | 1 (25%)  1 (25%)  1 (25%)  1 (25%) |
| **Activity tracker used in the intervention** | 0 | 0 | 2 (100%) | 2 (50%) |
| **Delivery mode**  Static  Adaptive (changing across the intervention based on participation) | 1 (100%)  0 | 0  1 (100%) | 0  2 (100%) | 1 (25%)  3 (75%) |

Note: *Very promising* = where significant increases in at least one sedentary behaviour indicator between the intervention group and the comparator arm. This excludes all single arm studies. *Quite promising* = where there were either significant changes in at least one sedentary behaviour outcome (for cohort studies) within the intervention group or when at least one sedentary behaviour outcome was improved but did not reach significance compared to one comparator arm (for multi-arm studies). *Non-promising* = where there were neither sedentary behaviour changes within the intervention arm nor differences relative to at least one comparator arm.
