# Supplementary material for: Smartphone applications for physical activity and sedentary behaviour change in people with cardiovascular disease: A systematic review and meta-analysis
Source: PLoS One. 2021 Oct 11;16(10):e0258460. doi: 10.1371/journal.pone.0258460 (PMC8504773; doi:10.1371/journal.pone.0258460)
Supplement: S4 Table — (DOCX) [file pone.0258460.s004.docx]

**S4 Table. Uptake, engagement, adherence and drop-out**

| **Study** | **Adherence and engagement to the app** | **Number of participants excluded or declined to participate** | **Drop-out rate** |
| --- | --- | --- | --- |
| Duscha, 2018a | No information | 338 excluded = 29.9% (101/338) no smartphone, 10.7% (36/338) already using an activity tracker, 6.8% (23/338) declined to participate, the rest were down to inability to exercise, medical issues, mobility issues, non-adherent to cardiac rehabilitation | Total = 20%  Exp = 3/16  Con = 2/9 |
| Duscha, 2018b | Adherence: 10% (1/10) met the prescribed 2,500 steps increase for at least 1-week in weeks 1-4.  10% (1/10) met the prescribed 3,750 steps increase for at least 1-week in weeks 5-8.  20% (2/10) met the prescribed 5,000 steps increase for at least 1-week in weeks 9-12.  Engagement: 70% (7/10) had attempted to increase steps as per intervention but did not meet the required 2,500 step increase at 1-4 weeks.  60% (6/10) had attempted to increase steps as per intervention but did not meet the required 3,750 step increase at 5-8 weeks.  50% (5/10) had attempted to increase steps as per intervention but did not meet the required 5,000 step increase at 5-8 weeks. | No information | Total = 5%  Exp = 0/10  Con = 1/10 |
| Freene, 2020 | Adherence: 73.7% (252/342) of the “Do” push notifications sent to participants were marked as complete | 40 excluded = 38% (15/40) declined to participate, 28% (11/40) did not have a smartphone and were significantly older than those with a smartphone (mean difference 20±5 yrs, p<0.001) | Total = 6-weeks 5%; 16-weeks 40%  Exp = 1/20; 8/20 |
| Grau-Pellicer, 2020 | Adherence: 50% (10/20) self-reported using the app | 150 excluded = reasons not provided | Total = 17%  Exp = 3/24  Con = 4/17 |
| Johnston, 2016 | Adherence: 82% of the intervention group and 83% of the control used e-diary for 150days | 8 excluded after starting = 75% (6/8) due to technical problems with the device, 25% (2/8) incorrectly randomised | Total = 2.4%  Exp = 1/86  Con = 3/80 |
| Kim, 2016 | Adherence: At 12-weeks, 70% (23/33) monitored their blood  pressure regularly at a recommended frequency  and at the end of the study  period, 55% (18/33) had still participated in the self-monitoring  program.  Engagement: Average BP measures recorded and synched by participants was beyond the encouraged amount at 104.9% (151/144) | No information | Total = unclear |
| Lunde, 2020 | Adherence: 71% (n=39) of patients answered all tasks throughout the year.  84% (n=46) answered more than 80% of the tasks and 91% (n=50) answered more than 50% of the tasks. | 64 excluded = 59.3% (38/64) not meeting inclusion criteria, 12.5% (8/64) not owner of smartphone, 21.9% (14/64) declined to participate, 4.7% (3/64) owner of smartphone unable to download the app, 1.6% (1/64) don’t want to use apps | Total = 1.8%  Exp = 2/57  Con = 0/56 |
| Lv, 2017 | Adherence: The number of participants meeting home BP monitoring frequency target (uploading 2x day and 3 days a week) started at ~87% (130/149) in week 1 and ~33% (50/149) at 6-months  Engagement: 44.3% did at least one of the 6 additional activities. | 1467 excluded = 35.9% (527/1467) declined participation, 9.7% (142/1467) were ineligible, 39.8% (584/1467) were not contactable or nonresponsive, 4.2% (62/1467) were interested but not enrolled as the study reached full enrollment before their initial study visit, 0.2% (3/1467) consented but did not complete baseline | Total = 1.3%  Exp = 2/149 |
| Nabutovsky, 2020 | Adherence: 63% attained the goal of 150 minutes of aerobic exercise per week.  Session duration training at or above target HR was 36.4%, which was less than the predefined study goal of 70%.  Adherence to two resistance training sessions per week was 18%.  Mobile app usage (the amount of time that the patients  used the mobile app) 3.7 ± 0.3, with a trend of higher usage at the third month compared to the other 5 months. | No information | Total = 4.5%  Exp = 1/22 |
| Paul, 2016 | No information | No information | Total = 4.2%  Exp = 1/16  Con = 0/8 |
| Persell, 2020 | Engagement: 37,346 conversations had with the AI app, 18,052 BP measures and 27,807 meals logged by 158 participants. | 2439 excluded = 18.5% (450/2439) no iOS phone, 29.6% (723/2439) refused, 39.4% (962/2439) not able to be contacted, 5.7% (140/2439) physician did not approve, 6.7% (164/2439) not meeting other inclusion criteria | Total = 10.8%  Exp = 22/166  Con = 14/167 |
| Requena, 2019 | No information | 298 excluded = excluded due to not being smartphone users before stroke (specified amount unclear) and other exclusion criteria. | Total = 0%  Exp = 0/107  Con = 0/52 |
| Salvi, 2018 | Adherence: Calculated by comparing the total number of prescribed exercise sessions with the number of sessions that were actually started by patients which was 61%.  Comparing the number of total performed minutes vs prescribed, adherence was only 32%  Engagement: The app sections most frequently accessed were those containing feedback including messages (31%), home (29%), exercise (18%), education (13%) and calendar (8%).  The percentage of exercise sessions explicitly cancelled by users was 4% | 14 excluded = reasons not provided | Total = 87.3%  Exp = 21-weeks 36/55; 6-months 47/55  Con = 21-weeks 21/63; 6-months 56/63 |
| Sengupta, 2020 | Engagement: Participants collectively set 132 goals (mean 16.5 ± 17.3) for a total of 3,335 minutes of walking and collectively achieved more than they intended (4,933 minutes).  The group responded to 830 EMA surveys and accessed 8 health educational videos 165 times (83% related to healthy eating behavior and 17% related to PA) | 1 excluded = reasons not provided | Total = 0%  Exp = 0/10 |
| Song, 2020 | No information | 27 excluded = 66.7% (18/27) refused to participate and 33.3% (9/27) fulfilled exclusion criteria | Total = 9.4%  Exp = 5/53  Con = 5/53 |
| Weerahandi, 2020 | Adherence: All participants logged their weight, steps, and dietary intake and received messages from the coach at some point across the study.  Engagement: mean number of blood pressure measurement = 63, mean number of weight measurement = 52, and mean number of daily steps logged = 55.  Most participants (15/17, 88%) utilized the chat feature to send messages to the coach and 16 participants (94%) logged their blood pressure and recorded goals.  Coaching phone calls had a high completion rate (74/102, 73%). | No information | Total = 0%  Exp = 0/17 |
| Werhahn, 2019 | Adherence: daily self-measured BP was 82.95%, 78.18% for body weight and 84.8% for compliance with wearing the Apple Watch.  More than half of the patients entered self-measured data on over 85% days of the study. | No information | Total = 10%  Exp = 1/10 |
| Widmer, 2015 | Engagement: Patient usage frequency was assessed by number of log in days divided by total number of active days and also assessed by logins per week with a higher frequency/percentage indicating higher patient participation. Average number of logins per week of 2.0 ±2.2 and average usage frequency of 44±28 % | No information | Total = unclear |
| Widmer, 2017 | Adherence: 6 patients (16%) in the digital health intervention group continued to use the app after the 3 months of the usual cardiac rehabilitation | 70 excluded = 95.7% (67/70) declined to participate, 4.3% (3/70) other reasons | Total = 0%  Exp = 0/37  Con = 0/34 |

Note abbreviations: App, Application; Con, Control group; Exp, Experimental group.
