# Supplementary material for: Smartphone applications for physical activity and sedentary behaviour change in people with cardiovascular disease: A systematic review and meta-analysis
Source: PLoS One. 2021 Oct 11;16(10):e0258460. doi: 10.1371/journal.pone.0258460 (PMC8504773; doi:10.1371/journal.pone.0258460)
Supplement: S1 File — (DOCX) [file pone.0258460.s005.docx]

**Supporting information 1 File: Full search strategy**

**P** – cardiovascular disease

**I** – smartphone applications (excluding interventions only using SMS text-messaging and phone calls)

**C** – usual care or lower functioning version of app

**O** – physical activity and sedentary behaviour (self-report or device-measured)

**S** – studies with a control group for the meta-analysis and cohort studies with pre-post outcomes for the narrative synthesis

Primary aim: To determine the influence of smartphone applications (excluding interventions only using SMS text-messages and phone calls) on physical activity and/or sedentary behaviour and their viability for use in people with cardiovascular disease.

Secondary aims:

- Participant characteristics and the factors which influence uptake (including requirement to own a smartphone), engagement and adherence to such interventions as well as retention rates.

Databases:

Medline, CINAHL Plus with Full Text (EBSCO), Cochrane Library, SCOPUS, Sports Discus, Embase

Time: 2007 to Current (31 October 2020)

Search terms:

| **Search Terms/Key Terms** | |
| --- | --- |
| Intervention:  Smartphone applications | 1. smartphone*; mobile device*; handheld device*; PDA; cellular phone*; cell phone*; mobile phone*; mobile-based; smartphone app*; mobile app*; tablet computer*; mobile health; mHealth; digital health; online health monitoring; |
| Outcomes:  Sedentary behaviours  Physical activity | 2. physical activity; exercise; aerobic exercise; physical fitness; fitness; active lifestyle; exercise capacity; exercise therapy; physical exertion; sedentar*; inactiv*; sedentary behaviour; sitting; sit time; screen time; sport*; walk*; movement; healthy lifestyle; |
| Population:  Cardiovascular disease | 3. cardiovascular disease; cardiovascular; cardiac rehabilitation; cardiac; coronary artery disease; coronary heart disease; coronary disease; hypertension; myocardial infarction; stroke; heart failure; arrhythmia; peripheral artery disease; cerebral vascular disease; heart; acute coronary syndrome; secondary prevention; |

(1 AND 2 AND 3)

**Database search strategies**

**Medline (EBSCOhost)**

MH “xxx” = Exact Subject Heading

MH “xxx+” = explode term

Near Operator (N) = N10 finds the words if they are within ten words of one another regardless of the order in which they appear

* = unlimited right-hand truncation

? = optional wild card stands for zero or one characters within a word or at the end of a word

| **Search term** | **Suggest Subject Terms** | **Database thesaurus information decision** |
| --- | --- | --- |
| smartphone* | Smartphone | (MH "Smartphone") |
| mobile device* |  | "mobile device*" |
| handheld device* | Computers, Handheld | (MH "Computers, Handheld+") |
| PDA | Computers, Handheld | "PDA"  Subject heading already included |
| cellular phone* | Cell Phone | (MH "Cell Phone+") |
| cell phone* | Cell Phone | Subject heading already included |
| mobile phone* | Cell Phone | Subject heading already included |
| mobile-based |  | Mobile-based |
| smartphone app* | Mobile Applications | (MH "Mobile Applications") OR "smartphone app*" |
| mobile app* | Mobile Applications | Subject heading already included |
| tablet computer* | Computers, Handheld | Subject heading already included |
| mobile health | Telemedicine | (MH "Telemedicine+") |
| mHealth | Telemedicine | "mHealth"  Subject heading already included |
| digital health |  | "digital health" |
| online health monitoring |  | online N10 health N10 monitoring |
| physical activity | Exercise  Fitness trackers | (MH "Exercise+") OR (MH "Fitness Trackers") OR "physical activity" |
| exercise | Exercise | Subject heading already included |
| aerobic exercise | Exercise | Subject heading already included |
| physical fitness | Physical Fitness | (MH "Physical Fitness+") |
| fitness | Fitness trackers | Subject heading already included |
| active lifestyle | Healthy Lifestyle  Leisure Activities  Human Activities | (MH "Healthy Lifestyle+") OR (MH "Leisure Activities+") OR (MH "Human Activities+") |
| exercise capacity | Exercise | Subject heading already included |
| exercise therapy | Exercise Therapy | (MH "Exercise Therapy+") |
| physical exertion | Physical Exertion | (MH "Physical Exertion") |
| sedentar* | Sedentary Behaviour | (MH "Sedentary Behavior") |
| physical inactiv* | Sedentary Behaviour | Subject heading already included |
| sedentary behaviour | Sedentary Behaviour | "Sedentary behavio?r"  Subject heading already included |
| sitting |  | "sitting" |
| sit time |  | "sit time" |
| screen time | Screen Time | (MH "Screen Time") |
| sport* | Sports | (MH "Sports+") |
| walk* | Walk Test | (MH "Walk Test") OR "walk*" |
| movement | Movement  Exercise Movement Techniques | (MH "Movement+") OR (MH "Exercise Movement Techniques+") |
| healthy lifestyle | Healthy Lifestyle  Healthy People Programs | (MH "Healthy Lifestyle+")  (MH "Healthy People Programs") |
| cardiovascular disease | Cardiovascular Diseases | (MH "Cardiovascular Diseases+") |
| cardiovascular | Cardiovascular Diseases | Subject heading already included |
| cardiac rehabilitation | Cardiac Rehabilitation  Stroke Rehabilitation | (MH "Cardiac Rehabilitation") OR (MH "Stroke Rehabilitation") |
| cardiac | Cardiac Rehabilitation | Subject heading already included |
| coronary artery disease | Coronary Artery Disease  Coronary Artery Bypass | (MH "Coronary Artery Disease") OR (MH "Coronary Artery Bypass+") |
| coronary heart disease | Coronary Disease  Heart Diseases  Myocardial Ischemia | (MH "Coronary Disease+") OR (MH "Heart Diseases+") OR (MH "Myocardial Ischemia+") |
| coronary disease | Coronary Disease | Subject heading already included |
| hypertension | Hypertension | (MH "Hypertension+") |
| myocardial infarction | Myocardial Infarction | (MH "Myocardial Infarction+") |
| stroke | Stroke | (MH "Stroke+") |
| heart failure | Heart Failure | (MH "Heart Failure+") |
| arrhythmia | Arrhythmias, Cardiac | (MH "Arrhythmias, Cardiac+") |
| peripheral artery disease | Peripheral Arterial Disease  Peripheral Vascular Diseases | (MH "Peripheral Arterial Disease") OR (MH "Peripheral Vascular Diseases+") |
| cerebral vascular disease | Cerebral Arterial Diseases | (MH "Cerebral Arterial Diseases+") |
| heart | Heart Diseases | Subject heading already included |
| acute coronary syndrome | Acute Coronary Syndrome | (MH "Acute Coronary Syndrome") |
| secondary prevention | Secondary Prevention | (MH "Secondary Prevention") |

| **Date of Search: 31/10/2020** | | | |
| --- | --- | --- | --- |
| **Search #** | **Concept** | **Search Terms** | **# of Results** |
| #1 | Intervention –  Smartphone applications | smartphone* OR (MH "Smartphone") OR "mobile device*" OR “handheld device*” OR (MH "Computers, Handheld+") OR "PDA" OR “cellular phone*” OR “cell phone*” OR “mobile phone*” OR (MH "Cell Phone+") OR mobile-based OR (MH "Mobile Applications") OR "smartphone app*" OR “mobile app*” OR (MH "Telemedicine+") OR “tablet computer*” OR “mobile health” OR "mHealth" OR "digital health" OR online N10 health N10 monitoring | 87,992 |
| #2 | Outcome –  Physical activity or Sedentary behaviour | (MH "Exercise+") OR (MH "Fitness Trackers") OR "physical activity" OR exercise OR “aerobic exercise” OR (MH "Physical Fitness+") OR “physical fitness” OR fitness OR “active lifestyle” OR (MH "Healthy Lifestyle+") OR (MH "Leisure Activities+") OR (MH "Human Activities+") OR (MH "Exercise Therapy+") OR “exercise therapy” OR “exercise capacity” OR “physical exertion” OR (MH "Physical Exertion") OR (MH "Sedentary Behavior") OR "Sedentary behavio?r" OR sedentar* OR inactiv* OR sitting OR "sit time" OR “screen time” OR (MH "Screen Time") OR sport* OR (MH "Sports+") OR (MH "Walk Test") OR "walk*" OR movement OR (MH "Movement+") OR (MH "Exercise Movement Techniques+") OR “healthy lifestyle”  OR (MH "Healthy Lifestyle+") OR (MH "Healthy People Programs") | 2,058,729 |
| #3 | Population –  Cardiovascular disease | “cardiovascular disease” OR (MH "Cardiovascular Diseases+") OR cardiovascular OR “cardiac rehabilitation” OR (MH "Cardiac Rehabilitation") OR cardiac OR (MH "Stroke Rehabilitation") OR “coronary artery disease” OR (MH "Coronary Artery Disease") OR (MH "Coronary Artery Bypass+") OR “coronary heart disease” OR “coronary disease” OR (MH "Coronary Disease+") OR (MH "Heart Diseases+") OR “myocardial infarction” OR (MH "Myocardial Ischemia+") OR hypertension OR (MH "Hypertension+") OR (MH "Myocardial Infarction+") OR stroke OR (MH "Stroke+") OR “heart failure” OR (MH "Heart Failure+") OR arrhythmia OR (MH "Arrhythmias, Cardiac+") OR (MH "Peripheral Arterial Disease") OR “peripheral artery disease” OR (MH "Peripheral Vascular Diseases+") OR “cerebral vascular disease” OR (MH "Cerebral Arterial Diseases+") OR heart OR “acute coronary syndrome” OR (MH "Acute Coronary Syndrome") OR “secondary prevention” OR (MH "Secondary Prevention") | 3,627,622 |
| #4 |  | #1 AND #2 AND #3 | 1893 |

**CINAHL Plus with Full Text (EBSCOhost)**

MH “xxx” = Exact Subject Heading

MH “xxx+” = explode term

Near Operator (N) = N10 finds the words if they are within ten words of one another regardless of the order in which they appear

* = unlimited right-hand truncation

? = optional wild card stands for zero or one characters within a word or at the end of a word

| **Search term** | **Suggest Subject Terms** | **Database thesaurus information decision** |
| --- | --- | --- |
| smartphone* | Smartphone | (MH "Smartphone") OR "smartphone*" |
| mobile device* |  | "mobile device*" |
| handheld device* |  | "handheld device*" |
| PDA | Computers, Hand-Held | (MH "Computers, Hand-Held+") OR "PDA" |
| cellular phone* | Cellular Phone | (MH "Cellular Phone+") |
| cell phone* | Cellular Phone | Subject heading already included |
| mobile phone* | Cellular Phone | Subject heading already included |
| mobile-based |  | "mobile-based" |
| smartphone app* | Mobile applications | (MH "Mobile Applications") OR "smartphone app*" |
| mobile app* | Mobile applications | “mobile app*”  Subject heading already included |
| tablet computer* | Computers, Portable | (MH "Computers, Portable+") |
| mobile health | Telehealth | (MH "Telehealth+") |
| mHealth | Telehealth | “mHealth”  Subject heading already included |
| digital health |  | "digital health" |
| online health monitoring |  | online N10 health N10 monitoring |
| physical activity | Physical Activity  Activities of Daily Living  Human Activities | (MH "Physical Activity") OR (MH "Activities of Daily Living+") OR (MH "Human Activities") |
| exercise | Exercise | (MH "Exercise+") |
| aerobic exercise | Aerobic Exercise | (MH "Aerobic Exercises+") |
| physical fitness | Physical Fitness | (MH "Physical Fitness+") |
| fitness | Physical Fitness | Subject heading already included |
| active lifestyle | Physical Activity  Activities of Daily Living | active N5 lifestyle  Subject heading already included |
| exercise capacity |  | “exercise capacity” |
| exercise therapy | Therapeutic Exercise | (MH "Therapeutic Exercise+") |
| physical exertion | Exertion | (MH "Exertion+") |
| sedentar* | Life Style, Sedentary | (MH "Life Style, Sedentary+") OR “sedentary*” |
| physical inactive* |  | "physical inactivit*" |
| sedentary behaviour | Life Style, Sedentary | sedentary behavio?r  Subject heading already included |
| sitting | Sitting | (MH "Sitting") |
| sit time | Sitting | "sit time"  Subject heading already included |
| screen time | Screen time | (MH "Screen Time") |
| sport* | Sports | (MH "Sports+") |
| walk* | Walking | (MH "Walking+") |
| movement | Movement | (MH "Movement+") |
| healthy lifestyle | Life Style Changes | (MH "Life Style Changes") OR "healthy lifestyle" |
| cardiovascular disease | Cardiovascular Diseases | (MH "Cardiovascular Diseases+") |
| cardiovascular | Diagnosis, Cardiovascular | (MH "Diagnosis, Cardiovascular+") |
| cardiac rehabilitation | Rehabilitation, Cardiac | (MH "Rehabilitation, Cardiac+") |
| cardiac | Cardiac patients | (MH "Cardiac Patients") |
| coronary artery disease | Coronary Arteriosclerosis  Coronary Artery Bypass | (MH "Coronary Arteriosclerosis") OR (MH "Coronary Artery Bypass+") |
| coronary heart disease | Coronary Disease  Heart Diseases | (MH "Coronary Disease+") OR (MH "Heart Diseases+") |
| coronary disease | Coronary Disease | Subject heading already included |
| hypertension | Hypertension | (MH "Hypertension+") |
| myocardial infarction | Myocardial Infarction | (MH "Myocardial Infarction") |
| stroke | Stroke  Stroke Patients | (MH "Stroke+") OR (MH "Stroke Patients") |
| heart failure | Heart Failure | (MH "Heart Failure+") |
| arrhythmia | Arrhythmia | (MH "Arrhythmia+") |
| peripheral artery disease | Peripheral Vascular Disease | (MH "Peripheral Vascular Diseases+") |
| cerebral vascular disease | Cerebrovascular Disorders | (MH "Cerebrovascular Disorders+") |
| heart | Heart | (MH "Heart+") |
| acute coronary syndrome | Acute Coronary Syndrome | (MH "Acute Coronary Syndrome") |
| secondary prevention | Preventative Healthcare | (MH "Preventive Health Care+") OR "secondary prevention" |

| **Date of Search: 31/10/2020** | | | |
| --- | --- | --- | --- |
| **Search #** | **Concept** | **Search Terms** | **# of Results** |
| #1 | Intervention –  Smartphone applications | (MH "Smartphone") OR "smartphone*" OR "mobile device*" OR "handheld device*" OR (MH "Computers, Hand-Held+") OR "PDA" OR “cellular phone*” OR “cell phone*” OR (MH "Cellular Phone+") OR “mobile phone*” OR "mobile-based" OR (MH "Mobile Applications") OR "smartphone app*" OR “mobile app*” OR “tablet computer*” OR (MH "Computers, Portable+") OR (MH "Telehealth+") OR “mobile health” OR “mHealth” OR "digital health" OR online N10 health N10 monitoring | 56,068 |
| #2 | Outcome –  Physical activity or Sedentary behaviour | “physical activity” OR (MH "Physical Activity") OR (MH "Activities of Daily Living+") OR (MH "Human Activities") OR exercise OR (MH "Exercise+") OR “aerobic exercise” OR (MH "Aerobic Exercises+") OR “physical fitness” OR (MH "Physical Fitness+") OR fitness OR active N5 lifestyle OR “exercise capacity” OR “exercise therapy” OR (MH "Therapeutic Exercise+") OR “physical exertion” OR (MH "Exertion+") OR (MH "Life Style, Sedentary+") OR “sedentar*” OR "physical inactivit*" OR sedentary behavio?r OR “sedentary behaviour” OR sitting OR (MH "Sitting") OR "sit time" OR “screen time” OR (MH "Screen Time") OR sport* OR (MH "Sports+") OR walk* OR (MH "Walking+") OR movement OR (MH "Movement+") OR (MH "Life Style Changes") OR "healthy lifestyle" | 585,150 |
| #3 | Population –  Cardiovascular disease | “cardiovascular disease” OR (MH "Cardiovascular Diseases+") OR cardiovascular OR (MH "Diagnosis, Cardiovascular+") OR “cardiac rehabilitation” OR (MH "Rehabilitation, Cardiac+") OR (MH "Cardiac Patients") OR cardiac OR (MH "Coronary Arteriosclerosis") OR “coronary artery disease” OR (MH "Coronary Artery Bypass+") OR (MH "Coronary Disease+") OR “coronary heart disease” OR “coronary disease” OR (MH "Heart Diseases+") OR hypertension OR (MH "Hypertension+") OR “myocardial infarction” OR (MH "Myocardial Infarction") OR stroke OR (MH "Stroke+") OR (MH "Stroke Patients") OR “heart failure” OR (MH "Heart Failure+") OR arrhythmia OR (MH "Arrhythmia+") OR “peripheral artery disease” OR (MH "Peripheral Vascular Diseases+") OR cerebral vascular disease OR (MH "Cerebrovascular Disorders+") OR heart OR (MH "Heart+") OR “acute coronary syndrome” OR (MH "Acute Coronary Syndrome") OR (MH "Preventive Health Care+") OR "secondary prevention" | 1,126,091 |
| #4 |  | #1 AND #2 AND #3 | 1,153 |

**Cochrane Central Register of Controlled Trials (CENTRAL)**

NEAR = finds the terms when they are within 6 words of each other

* = unlimited right-hand truncation

? = optional wild card stands for zero or one characters within a word or at the end of a word

[mh XXXXXXXXX] = MeSH heading with term explosion

| **Search term** | **Map term to subject heading** | **Database thesaurus information decision** |
| --- | --- | --- |
| smartphone* | Smartphone | [mh Smartphone] |
| mobile device* |  | "mobile device*" |
| handheld device* | Computers, Handheld | [mh "Computers, Handheld"] |
| PDA | Computers, Handheld | PDA  Subject heading already included |
| cellular phone* | Cell Phone | [mh "Cell Phone"] |
| cell phone* | Cell Phone | Subject heading already included |
| mobile phone* | Cell Phone | “mobile phone*”  Subject heading already included |
| mobile-based |  | mobile-based |
| smartphone app* | Smartphone | “smartphone app*”  Subject heading already included |
| mobile app* | Mobile Applications | [mh “Mobile Applications”] |
| tablet computer* | Computers, Handheld | “tablet computer*”  Subject heading already included |
| mobile health | Telemedicine | [mh Telemedicine] |
| mHealth | Telemedicine | “mHealth”  Subject heading already included |
| digital health |  | digital NEAR health |
| online health monitoring |  | online NEAR health NEAR monitoring |
| physical activity | Exercise  Fitness Trackers  Human Activities  Leisure Activities | [mh Exercise] OR [mh “Fitness Trackers”] OR [mh “Human Activities”] OR [mh “Leisure Activities”] OR “Physical Activity” |
| exercise | Exercise | Subject heading already included |
| aerobic exercise | Exercise | Subject heading already included |
| physical fitness | Physical Fitness | [mh “Physical Fitness”] |
| fitness | Cardiorespiratory Fitness | [mh “Cardiorespiratory Fitness”] |
| active lifestyle | Healthy Lifestyle  Life Style | [mh “Healthy Lifestyle”] OR [mh “Life Style”] |
| exercise capacity | Exercise | “Exercise Capacity”  Subject heading already included |
| exercise therapy | Exercise Therapy | [mh “Exercise Therapy”] |
| physical exertion | Physical Exertion | [mh “Physical Exertion”] |
| sedentar* | Sedentary Behavior | [mh “Sedentary Behavior”] OR sedentar* |
| physical inactiv* |  | physical inactiv* |
| sedentary behaviour | Sedentary Behavior | “Sedentary Behaviour”  Subject heading already included |
| sitting |  | sitting |
| sit time |  | “sit time” |
| screen time | Screen Time | [mh “Screen Time”] |
| sport* | Sports Medicine | [mh “Sports Medicine”] |
| walk* | Walking  Walk Test | [mh walking] OR [mh “walk test”] |
| movement | Movement | [mh movement] |
| healthy lifestyle | Healthy Lifestyle  Healthy People Programs | [mh “Healthy Lifestyle”]  [mh “Healthy People Programs”] |
| cardiovascular disease | Cardiovascular Diseases | [mh “Cardiovascular Diseases”] |
| cardiovascular | Cardiology | [mh cardiology] |
| cardiac rehabilitation | Cardiac Rehabilitation  Stroke Rehabilitation | [mh “Cardiac Rehabilitation”] OR [mh “Stroke Rehabilitation”] |
| cardiac | Cardiac Rehabilitation | Subject heading already included |
| coronary artery disease | Coronary Artery Disease  Coronary Disease  Coronary Artery Bypass | [mh “Coronary Artery Disease”] OR [mh “Coronary Disease”] OR [mh “Coronary Artery Bypass”] |
| coronary heart disease | Heart Diseases  Myocardial Ischemia | [mh “Heart Diseases”] OR [mh “Myocardial Ischemia”] |
| coronary disease | Coronary Disease | [mh “Coronary Disease”] |
| hypertension | Hypertension | [mh Hypertension] |
| myocardial infarction | Myocardial Infarction | [mh “Myocardial Infarction”] |
| stroke | Stroke | [mh Stroke] |
| heart failure | Heart Failure | [mh “Heart Failure”] |
| arrhythmia | Arrhythmia, Cardiac | [mh “Arrhythmia, Cardiac”] |
| peripheral artery disease | Peripheral Artery Disease  Peripheral Vascular Diseases | [mh “Peripheral Artery Disease”] OR [mh “Peripheral Vascular Diseases”] |
| cerebral vascular disease | Cerebral Arterial Diseases | [mh “Cerebral Arterial Diseases”] |
| heart | Heart Diseases | Subject heading already included |
| acute coronary syndrome | Acute Coronary Syndrome | [mh “Acute Coronary Syndrome”] |
| secondary prevention | Secondary Prevention | [mh “Secondary Prevention”] |

| **Date of Search: 31/10/2020** | | | |
| --- | --- | --- | --- |
| **Search #** | **Concept** | **Search Terms** | **# of Results** |
| #1 | Intervention –  Smartphone applications | smartphone* OR [mh Smartphone] OR "mobile device*" OR “handheld device*” OR [mh "Computers, Handheld"] OR PDA OR “cellular phone*” OR “cell phone*” OR [mh "Cell Phone"] OR “mobile phone*” OR mobile-based OR “smartphone app*” OR “mobile app*” OR [mh “Mobile Applications”] OR “tablet computer*” OR “mobile health” OR [mh Telemedicine] OR “mHealth” OR digital NEAR health OR online NEAR health NEAR monitoring | 12,875 |
| #2 | Outcome –  Physical activity or Sedentary behaviour | [mh Exercise] OR [mh “Fitness Trackers”] OR [mh “Human Activities”] OR [mh “Leisure Activities”] OR “Physical Activity” OR “physical fitness” OR [mh “Physical Fitness”] OR “aerobic exercise” OR [mh “Cardiorespiratory Fitness”] OR fitness OR [mh “Healthy Lifestyle”] OR “active lifestyle” OR [mh “Life Style”] OR “Exercise Capacity” OR [mh “Exercise Therapy”] OR “exercise therapy” OR [mh “Physical Exertion”] OR “physical exertion” OR [mh “Sedentary Behavior”] OR sedentar* OR “physical inactiv*” OR “Sedentary Behaviour” OR sitting OR “sit time” OR [mh “Screen Time”] OR “screen time” OR sport* OR [mh “Sports Medicine”] OR walk* OR [mh walking] OR [mh “walk test”] OR movement OR [mh movement] OR “healthy lifestyle” OR [mh “Healthy Lifestyle”] OR [mh “Healthy People Programs”] | 153,912 |
| #3 | Population –  Cardiovascular disease | [mh “Cardiovascular Diseases”] OR “cardiovascular disease” OR cardiovascular OR [mh cardiology] OR “cardiac rehabilitation” OR [mh “Cardiac Rehabilitation”] OR [mh “Stroke Rehabilitation”] OR cardiac OR “coronary artery disease” OR [mh “Coronary Artery Disease”] OR [mh “Coronary Disease”] OR “coronary disease” OR [mh “Coronary Artery Bypass”] OR “coronary heart disease” OR [mh “Heart Diseases”] OR [mh “Myocardial Ischemia”] OR hypertension OR [mh Hypertension] OR “myocardial infarction” OR [mh “Myocardial Infarction”] OR stroke OR [mh Stroke] OR “heart failure” OR [mh “Heart Failure”] OR arrhythmia OR [mh “Arrhythmia, Cardiac”] OR “peripheral artery disease” OR [mh “Peripheral Artery Disease”] OR [mh “Peripheral Vascular Diseases”] OR “cerebral vascular disease” OR [mh “Cerebral Arterial Diseases”] OR heart OR “acute coronary syndrome” OR [mh “Acute Coronary Syndrome”] OR “secondary prevention” OR [mh “Secondary Prevention”] | 335,564 |
| #4 |  | #1 AND #2 AND #3 | 612 |

SCOPUS

* = unlimited right-hand truncation

? = optional wild card stands for zero or one characters within a word or at the end of a word

| **Search term** | **Map term to subject heading: N/A** | **Database thesaurus information decision** |
| --- | --- | --- |
| smartphone* |  | Smartphone* |
| mobile device* |  | “mobile device*” |
| handheld device* |  | “handheld device*” |
| PDA |  | PDA |
| cellular phone* |  | “cellular phone*” |
| cell phone* |  | “cell phone*” |
| mobile phone* |  | “mobile phone*” |
| mobile-based |  | mobile-based |
| smartphone app* |  | “smartphone app*” |
| mobile app* |  | “mobile app*” |
| tablet computer* |  | “tablet computer*” |
| mobile health |  | “mobile health” |
| mHealth |  | mHealth |
| digital health |  | “digital health” |
| online health monitoring |  | “online health monitoring” |
| physical activity |  | “physical activity” |
| exercise |  | exercise |
| aerobic exercise |  | “aerobic exercise” |
| physical fitness |  | “physical fitness” |
| fitness |  | fitness |
| active lifestyle |  | “active lifestyle” |
| exercise capacity |  | “exercise capacity” |
| exercise therapy |  | “exercise therapy” |
| physical exertion |  | “physical exertion” |
| sedentar* |  | Sedentar* |
| physical inactiv* |  | “physical inactiv*” |
| sedentary behaviour |  | “sedentary behavior” |
| sitting |  | sitting |
| sit time |  | “sit time” |
| screen time |  | “screen time” |
| sport* |  | sport* |
| walk* |  | walk* |
| movement |  | movement |
| healthy lifestyle |  | “healthy lifestyle” |
| cardiovascular disease |  | “cardiovascular disease” |
| cardiovascular |  | cardiovascular |
| cardiac rehabilitation |  | “cardiac rehabilitation” |
| cardiac |  | cardiac |
| coronary artery disease |  | “coronary artery disease” |
| coronary heart disease |  | “coronary heart disease” |
| coronary disease |  | “coronary disease” |
| hypertension |  | hypertension |
| myocardial infarction |  | “myocardial infarction” |
| stroke |  | stroke |
| heart failure |  | “heart failure” |
| arrhythmia |  | arrhythmia |
| peripheral artery disease |  | “peripheral artery disease” |
| cerebral vascular disease |  | “cerebral vascular disease” |
| Heart disease |  | “heart disease” |
| acute coronary syndrome |  | “acute coronary syndrome” |
| secondary prevention |  | “secondary prevention” |

| **Date of Search: 31/10/2020** | | | |
| --- | --- | --- | --- |
| **Search #** | **Concept** | **Search Terms** | **# of Results** |
| #1 | Intervention –  Smartphone applications | TITLE-ABS(Smartphone* OR “mobile device*” OR “handheld device*” OR PDA OR “cellular phone*” OR “cell phone*” OR “mobile phone*” OR mobile-based OR “smartphone app*” OR “mobile app*” OR “tablet computer*” OR “mobile health” OR mHealth OR “digital health” OR “online health monitoring”) | 261,256 |
| #2 | Outcome –  Physical activity or Sedentary behaviour | TITLE-ABS(“physical activity” OR exercise OR “aerobic exercise” OR “physical fitness” OR fitness OR “active lifestyle” OR “exercise capacity” OR “exercise therapy” OR “physical exertion” OR Sedentar* OR “physical inactiv*” OR “sedentary behavior” OR sitting OR “sit time” OR “screen time” OR walk* OR “healthy lifestyle”) | 1,218,343 |
| #3 | Population –  Cardiovascular disease | TITLE-ABS(“cardiovascular disease” OR cardiovascular OR “cardiac rehabilitation” OR cardiac OR “coronary artery disease” OR “coronary heart disease” OR “coronary disease” OR hypertension OR “myocardial infarction” OR stroke OR “heart failure” OR arrhythmia OR “peripheral artery disease” OR “cerebral vascular disease” OR “heart disease” OR “acute coronary syndrome” OR “secondary prevention”) | 3,019,748 |
| #4 |  | #1 AND #2 AND #3 | 1,046 |

SPORTDiscuss with Full Text (EBSCOhost)

(DE XXXX) = Exact Subject Heading

Near Operator (N) = N10 finds the words if they are within ten words of one another regardless of the order in which they appear

* = unlimited right-hand truncation

? = optional wild card stands for zero or one characters within a word or at the end of a word

| **Search term** | **Sports Thesaurus Terms** | **Database thesaurus information decision** |
| --- | --- | --- |
| smartphone* |  | Smartphone* |
| mobile device* |  | “mobile device*” |
| handheld device* |  | “handheld device*” |
| PDA |  | PDA |
| cellular phone* |  | “cellular phone*” |
| cell phone* |  | “cell phone*” |
| mobile phone* |  | “mobile phone*” |
| mobile-based |  | mobile-based |
| smartphone app* |  | “smartphone app*” |
| mobile app* |  | “mobile app*” |
| tablet computer* |  | “tablet computer*” |
| mobile health |  | “mobile health” |
| mHealth |  | mHealth |
| digital health |  | “digital health” |
| online health monitoring |  | “online health monitoring” |
| physical activity | PHYSICAL activity | (DE "PHYSICAL activity") |
| exercise | EXERCISE | (DE "EXERCISE" OR DE "ABDOMINAL exercises" OR DE "AEROBIC exercises" OR DE "ANAEROBIC exercises" OR DE "AQUATIC exercises" OR DE "ARM exercises" OR DE "BACK exercises" OR DE "BREATHING exercises" OR DE "BREEMA" OR DE "BUTTOCKS exercises" OR DE "CALISTHENICS" OR DE "CHAIR exercises" OR DE "CHEST exercises" OR DE "CIRCUIT training" OR DE "COMPOUND exercises" OR DE "COOLDOWN" OR DE "DO-in" OR DE "EXERCISE adherence" OR DE "EXERCISE for children" OR DE "EXERCISE for girls" OR DE "EXERCISE for men" OR DE "EXERCISE for middle-aged persons" OR DE "EXERCISE for older people" OR DE "EXERCISE for people with disabilities" OR DE "EXERCISE for women" OR DE "EXERCISE for youth" OR DE "EXERCISE therapy" OR DE "EXERCISE video games" OR DE "FACIAL exercises" OR DE "FALUN gong exercises" OR DE "FOOT exercises" OR DE "GYMNASTICS" OR DE "HAND exercises" OR DE "HATHA yoga" OR DE "HIP exercises" OR DE "ISOKINETIC exercise" OR DE "ISOLATION exercises" OR DE "ISOMETRIC exercise" OR DE "ISOTONIC exercise" OR DE "KNEE exercises" OR DE "LEG exercises" OR DE "LIANGONG" OR DE "METABOLIC equivalent" OR DE "MULAN quan" OR DE "MUSCLE strength" OR DE "PILATES method" OR DE "PLYOMETRICS" OR DE "QI gong" OR DE "REDUCING exercises" OR DE "RUNNING" OR DE "RUNNING -- Social aspects" OR DE "SCHOOL exercises & recreations" OR DE "SEXUAL exercises" OR DE "SHOULDER exercises" OR DE "STRENGTH training" OR DE "STRESS management exercises" OR DE "TAI chi" OR DE "TREADMILL exercise" OR DE "WHEELCHAIR workouts" OR DE "YOGA") |
| aerobic exercise | EXERCISE | Subject heading already included |
| physical fitness | PHYSICAL fitness | (DE "PHYSICAL fitness testing" OR DE "EUROFIT" OR DE "HEART function tests" OR DE "JUMP & reach tests" OR DE "KRAUS-Weber test" OR DE "MUSCLE strength measurement" OR DE "PHYSICAL Activity Readiness Questionnaire" OR DE "PHYSICAL fitness testing for children" OR DE "STEP tests") |
| fitness | CARDIOVASCULAR fitness | DE "CARDIOVASCULAR fitness" |
| active lifestyle | LIFESTYLES  PHYSICALLY active people  LEISURE | (DE "LIFESTYLES" OR DE "SEDENTARY lifestyles") OR (DE "PHYSICALLY active people") OR (DE "LEISURE" OR DE "AMATEURISM" OR DE "RETIREMENT") |
| exercise capacity | AEROBIC capacity | DE "AEROBIC capacity" |
| exercise therapy | EXERCISE therapy | (DE "EXERCISE therapy" OR DE "EXERCISE therapy for children" OR DE "EXERCISE therapy for older people" OR DE "MENSENDIECK system" OR DE "ORTHOPTICS" OR DE "SWEDISH gymnastics" OR DE "THERAPEUTIC use of breathing exercises") |
| physical exertion | PHYSICAL fitness | Subject heading already included |
| sedentar* | SEDENTARY lifestyles  SEDENTARY people | (DE "SEDENTARY lifestyles") OR (DE "SEDENTARY people" OR DE "SEDENTARY women") |
| physical inactiv* | SEDENTARY behavior | DE "SEDENTARY behavior" |
| sedentary behaviour | SEDENTARY behavior | Subject heading already included |
| sitting |  | sitting |
| sit time |  | “sit time” |
| screen time |  | “screen time” |
| sport* |  | Sport* |
| walk* | FITNESS walking  WALKING | (DE "FITNESS walking") OR (DE "WALKING" OR DE "FITNESS walking" OR DE "GAIT in humans" OR DE "HIKING" OR DE "LONG distance walking" OR DE "VIERDAAGSE (Walking event)") |
| movement |  | movement |
| healthy lifestyle | LIFESTYLES | Subject heading already included |
| cardiovascular disease | CARDIOVASCULAR diseases | DE "CARDIOVASCULAR diseases" OR DE "BLOOD circulation disorders" OR DE "HEART diseases" OR DE "HYPERTENSION" OR DE "THROMBOSIS" OR DE "UPPER extremity deep vein thrombosis" OR DE "VASCULAR diseases" |
| cardiovascular | CARDIOVASCULAR diseases | Subject heading already included |
| cardiac rehabilitation | REHABILITATION | DE "REHABILITATION" OR DE "AQUATIC exercises -- Therapeutic use" OR DE "MEDICAL rehabilitation" OR DE "NEUROPSYCHOLOGICAL rehabilitation" |
| cardiac | CARDIAC arrest  CARDIAC patients | (DE "CARDIAC arrest" OR DE "CARDIAC resuscitation" OR DE "VENTRICULAR fibrillation") OR DE "CARDIAC patients" |
| coronary artery disease | CORONARY disease  CORONARY artery bypass | (DE "CORONARY disease" OR DE "ANGINA pectoris" OR DE "MYOCARDIAL infarction") OR (DE "CORONARY artery bypass") |
| coronary heart disease | HEART diseases | DE "HEART diseases" OR DE "ARRHYTHMIA" OR DE "CARDIAC arrest" OR DE "CARDIAC hypertrophy" OR DE "CARDIOMYOPATHIES" OR DE "CORONARY disease" OR DE "HEART block" OR DE "HEART dilatation" OR DE "HEART failure" |
| coronary disease | CORONARY disease | Subject heading already included |
| hypertension | HYPERTENSION | DE "HYPERTENSION" OR DE "ANTIHYPERTENSIVE agents" OR DE "PULMONARY hypertension" |
| myocardial infarction | MYOCARDIAL infarction | DE "MYOCARDIAL infarction" |
| stroke | STROKE | DE "STROKE" |
| heart failure | HEART failure | DE "HEART failure" OR DE "CONGESTIVE heart failure" |
| arrhythmia | ARRHYTHMIA | DE "ARRHYTHMIA" OR DE "ARRHYTHMIA treatment" OR DE "ATRIAL arrhythmias" OR DE "BRADYCARDIA" OR DE "EXTRASYSTOLE" OR DE "HEART block" OR DE "SINUS arrhythmia" OR DE "TACHYCARDIA" OR DE "VENTRICULAR fibrillation" OR DE "WOLFF-Parkinson-White syndrome" |
| peripheral artery disease | PERIPHERAL vascular diseases | DE "PERIPHERAL vascular diseases" OR DE "HYPOTHENAR hammer syndrome" OR DE "RAYNAUD'S disease" |
| cerebral vascular disease | CEREBROVASCULAR disease  CEREBROVASCULAR disease patients | (DE "CEREBROVASCULAR disease" OR DE "CEREBRAL embolism & thrombosis" OR DE "CEREBRAL hemorrhage" OR DE "CLUSTER headache" OR DE "STROKE") OR (DE "CEREBROVASCULAR disease patients" OR DE "STROKE patients") |
| heart | HEART diseases | Subject heading already included |
| acute coronary syndrome | CORONARY disease | Subject heading already included |
| secondary prevention | PREVENTATIVE medicine | DE "PREVENTIVE medicine" OR DE "HEALTH risk assessment" OR DE "MEDICAL self-examination" OR DE "PERIODIC health examinations" |

| **Date of Search: 31/10/2020** | | | |
| --- | --- | --- | --- |
| **Search #** | **Concept** | **Search Terms** | **# of Results** |
| #1 | Intervention –  Smartphone applications | Smartphone* OR “mobile device*” OR “handheld device*” OR PDA OR “cellular phone*” OR “cell phone*” OR “mobile phone*” OR mobile-based OR “smartphone app*” OR “mobile app*” OR “tablet computer*” OR “mobile health” OR mHealth OR “digital health” OR “online health monitoring” | 5,152 |
| #2 | Outcome –  Physical activity or Sedentary behaviour | “physical activity” OR DE "PHYSICAL activity" OR exercise OR DE "EXERCISE" OR DE "ABDOMINAL exercises" OR “aerobic exercise” OR DE "AEROBIC exercises" OR DE "ANAEROBIC exercises" OR DE "AQUATIC exercises" OR DE "ARM exercises" OR DE "BACK exercises" OR DE "BREATHING exercises" OR DE "BREEMA" OR DE "BUTTOCKS exercises" OR DE "CALISTHENICS" OR DE "CHAIR exercises" OR DE "CHEST exercises" OR DE "CIRCUIT training" OR DE "COMPOUND exercises" OR DE "COOLDOWN" OR DE "DO-in" OR DE "EXERCISE adherence" OR DE "EXERCISE for children" OR DE "EXERCISE for girls" OR DE "EXERCISE for men" OR DE "EXERCISE for middle-aged persons" OR DE "EXERCISE for older people" OR DE "EXERCISE for people with disabilities" OR DE "EXERCISE for women" OR DE "EXERCISE for youth" OR DE "EXERCISE therapy" OR DE "EXERCISE video games" OR DE "FACIAL exercises" OR DE "FALUN gong exercises" OR DE "FOOT exercises" OR DE "GYMNASTICS" OR DE "HAND exercises" OR DE "HATHA yoga" OR DE "HIP exercises" OR DE "ISOKINETIC exercise" OR DE "ISOLATION exercises" OR DE "ISOMETRIC exercise" OR DE "ISOTONIC exercise" OR DE "KNEE exercises" OR DE "LEG exercises" OR DE "LIANGONG" OR DE "METABOLIC equivalent" OR DE "MULAN quan" OR DE "MUSCLE strength" OR DE "PILATES method" OR DE "PLYOMETRICS" OR DE "QI gong" OR DE "REDUCING exercises" OR DE "RUNNING" OR DE "RUNNING -- Social aspects" OR DE "SCHOOL exercises & recreations" OR DE "SEXUAL exercises" OR DE "SHOULDER exercises" OR DE "STRENGTH training" OR DE "STRESS management exercises" OR DE "TAI chi" OR DE "TREADMILL exercise" OR DE "WHEELCHAIR workouts" OR DE "YOGA" OR “physical fitness” OR fitness OR DE "PHYSICAL fitness testing" OR DE "EUROFIT" OR DE "HEART function tests" OR DE "JUMP & reach tests" OR DE "KRAUS-Weber test" OR DE "MUSCLE strength measurement" OR DE "PHYSICAL Activity Readiness Questionnaire" OR DE "PHYSICAL fitness testing for children" OR DE "STEP tests" OR DE "CARDIOVASCULAR fitness" OR “active lifestyle” OR DE "LIFESTYLES" OR DE "SEDENTARY lifestyles" OR DE "PHYSICALLY active people" OR DE "LEISURE" OR DE "AMATEURISM" OR DE "RETIREMENT" OR “exercise capacity” OR DE "AEROBIC capacity" OR “exercise therapy” OR DE "EXERCISE therapy" OR DE "EXERCISE therapy for children" OR DE "EXERCISE therapy for older people" OR DE "MENSENDIECK system" OR DE "ORTHOPTICS" OR DE "SWEDISH gymnastics" OR DE "THERAPEUTIC use of breathing exercises" OR “physical exertion” OR sedentar* OR inactiv* OR “sedentary behaviour” OR DE "SEDENTARY lifestyles" OR DE "SEDENTARY people" OR DE "SEDENTARY women" OR DE "SEDENTARY behavior" OR sitting OR “sit time” OR “screen time” OR Sport* OR walk* OR DE "FITNESS walking" OR DE "WALKING" OR DE "FITNESS walking" OR DE "GAIT in humans" OR DE "HIKING" OR DE "LONG distance walking" OR DE "VIERDAAGSE (Walking event)" OR movement OR “healthy lifestyle” | 1,312,358 |
| #3 | Population –  Cardiovascular disease | “cardiovascular disease” OR cardiovascular OR DE "CARDIOVASCULAR diseases" OR DE "BLOOD circulation disorders" OR “coronary artery disease” OR “coronary heart disease” OR DE "HEART diseases" OR DE "HYPERTENSION" OR DE "THROMBOSIS" OR DE "UPPER extremity deep vein thrombosis" OR DE "VASCULAR diseases" OR “cardiac rehabilitation” OR cardiac OR DE "REHABILITATION" OR DE "AQUATIC exercises -- Therapeutic use" OR DE "MEDICAL rehabilitation" OR DE "NEUROPSYCHOLOGICAL rehabilitation" OR DE "CARDIAC arrest" OR DE "CARDIAC resuscitation" OR DE "VENTRICULAR fibrillation" OR DE "CARDIAC patients" OR DE "CORONARY disease" OR DE "ANGINA pectoris" OR DE "MYOCARDIAL infarction" OR DE "CORONARY artery bypass" OR DE "HEART diseases" OR DE "ARRHYTHMIA" OR DE "CARDIAC arrest" OR DE "CARDIAC hypertrophy" OR DE "CARDIOMYOPATHIES" OR “coronary disease” OR DE "CORONARY disease" OR DE "HEART block" OR DE "HEART dilatation" OR “heart failure” OR DE "HEART failure" OR hypertension OR DE "HYPERTENSION" OR DE "ANTIHYPERTENSIVE agents" OR DE "PULMONARY hypertension" OR “myocardial infarction” OR DE "MYOCARDIAL infarction" OR DE "STROKE" OR DE "HEART failure" OR DE "CONGESTIVE heart failure" OR arrhythmia OR DE "ARRHYTHMIA" OR DE "ARRHYTHMIA treatment" OR DE "ATRIAL arrhythmias" OR DE "BRADYCARDIA" OR DE "EXTRASYSTOLE" OR DE "HEART block" OR DE "SINUS arrhythmia" OR DE "TACHYCARDIA" OR DE "VENTRICULAR fibrillation" OR DE "WOLFF-Parkinson-White syndrome" OR “peripheral artery disease” OR DE "PERIPHERAL vascular diseases" OR DE "HYPOTHENAR hammer syndrome" OR DE "RAYNAUD'S disease" OR “cerebral vascular disease” OR DE "CEREBROVASCULAR disease" OR DE "CEREBRAL embolism & thrombosis" OR DE "CEREBRAL hemorrhage" OR DE "CLUSTER headache" OR stroke OR DE "STROKE" OR DE "CEREBROVASCULAR disease patients" OR DE "STROKE patients" OR heart OR “acute coronary syndrome” OR DE "PREVENTIVE medicine" OR DE "HEALTH risk assessment" OR DE "MEDICAL self-examination" OR DE "PERIODIC health examinations" OR “secondary prevention” | 147,778 |
| #4 |  | #1 AND #2 AND #3 | 169 |

Embase (Ovid)

* = unlimited right-hand truncation

| **Search term** | **Map term to subject heading:** | **Database thesaurus information decision** |
| --- | --- | --- |
| smartphone* | Exp smartphone/ | exp smartphone/ or smartphone*.mp. |
| mobile device* | Exp Mobile phone/ | exp mobile phone/ or mobile device*.mp. |
| handheld device* |  | handheld device*.mp. |
| PDA | exp personal digital assistant/ | exp personal digital assistant/ or PDA.mp. |
| cellular phone* |  | cellular phone*.mp. |
| cell phone* |  | cell phone*.mp. |
| mobile phone* | Exp Mobile phone/ OR exp telephone/ | mobile phone*.mp. or exp telephone/ or exp mobile phone/ |
| mobile-based |  | mobile-based.mp. |
| smartphone app* | exp mobile application/ | smartphone app*.mp. or exp mobile application/ |
| mobile app* | exp mobile application/ | mobile app*.mp. or exp mobile application/ |
| tablet computer* | exp tablet computer/ | tablet computer*.mp. or exp tablet computer/ |
| mobile health |  | mobile health.mp. |
| mHealth |  | mHealth.mp. |
| digital health |  | digital health.mp. |
| online health monitoring | exp patient monitoring/ | online health monitoring.mp. or exp patient monitoring/ |
| physical activity | exp physical activity/ | physical activity.mp. or exp physical activity/ |
| exercise | exp exercise/ | exp exercise/ or exercise.mp. |
| aerobic exercise | exp aerobic exercise/ | aerobic exercise.mp. or exp aerobic exercise/ |
| physical fitness | exp fitness/ | physical fitness.mp. or exp fitness/ |
| fitness | exp fitness/ | exp fitness/ or fitness.mp. |
| active lifestyle |  | active lifestyle.mp. |
| exercise capacity | exp exercise/ | exercise capacity.mp. or exp exercise/ |
| exercise therapy | exp kinesiotherapy/ | exercise therapy.mp. or exp kinesiotherapy/ |
| physical exertion | exp exercise/ | physical exertion.mp. or exp exercise/ |
| sedentar* | exp sedentary lifestyle/ | exp sedentary lifestyle/ or sedentar*.mp. |
| physical inactiv* | exp physical inactivity/ | exp physical inactivity/ or physical inactiv*.mp. |
| sedentary behaviour | exp sedentary lifestyle/ | sedentary behaviour.mp. or exp sedentary lifestyle/ |
| sitting | exp sitting/ | sitting.mp. or exp sitting/ |
| sit time |  | sit time.mp. |
| screen time | exp screen time/ | screen time.mp. or exp screen time/ |
| sport* | exp sport/ | sport*.mp. or exp sport/ |
| walk* | exp walk test/ | exp walk test/ or walk*.mp. |
| movement | exp "movement (physiology)"/ | movement.mp. or exp "movement (physiology)"/ |
| healthy lifestyle | exp healthy lifestyle/ or exp health behavior/ or exp health promotion/ | healthy lifestyle.mp. or exp healthy lifestyle/ or exp health behavior/ or exp health promotion/ |
| cardiovascular disease | exp cardiovascular disease/ | cardiovascular disease.mp. or exp cardiovascular disease/ |
| cardiovascular | exp cardiovascular disease/ | exp cardiovascular disease/ or cardiovascular.mp. |
| cardiac rehabilitation | exp heart rehabilitation/ | cardiac rehabilitation.mp. or exp heart rehabilitation/ |
| cardiac | exp cardiac patient/ | exp cardiac patient/ or cardiac.mp. |
| coronary artery disease | exp coronary artery disease/ | coronary artery disease.mp. or exp coronary artery disease/ |
| coronary heart disease | exp ischemic heart disease/ | coronary heart disease.mp. or exp ischemic heart disease/ |
| coronary disease | coronary disease.mp. or exp coronary artery disease/ | coronary disease.mp. or exp coronary artery disease/ |
| hypertension | exp hypertension/ | exp hypertension/ or hypertension.mp. |
| myocardial infarction | exp heart infarction/ | myocardial infarction.mp. or exp heart infarction/ |
| stroke | exp cerebrovascular accident/ | stroke.mp. or exp cerebrovascular accident/ |
| heart failure | exp heart failure/ | heart failure.mp. or exp heart failure/ |
| arrhythmia | exp heart arrhythmia/ | arrhythmia.mp. or exp heart arrhythmia/ |
| peripheral artery disease | exp peripheral occlusive artery disease/ | peripheral artery disease.mp. or exp peripheral occlusive artery disease/ |
| cerebral vascular disease | exp cerebrovascular disease/ | cerebral vascular disease.mp. or exp cerebrovascular disease/ |
| Heart disease | exp heart disease/ | Heart disease.mp. or exp heart disease/ |
| acute coronary syndrome | exp acute coronary syndrome/ | acute coronary syndrome.mp. or exp acute coronary syndrome/ |
| secondary prevention | exp secondary prevention/ | secondary prevention.mp. or exp secondary prevention/ |

| **Date of Search: 31/10/2020** | | | |
| --- | --- | --- | --- |
| **Search #** | **Concept** | **Search Terms** | **# of Results** |
| #1 | Intervention –  Smartphone applications | exp smartphone/ or smartphone*.mp. OR exp mobile phone/ or mobile device*.mp. OR handheld device*.mp. OR exp personal digital assistant/ or PDA.mp. OR cellular phone*.mp. OR cell phone*.mp. OR mobile phone*.mp. or exp telephone/ or mobile-based.mp. OR smartphone app*.mp. or exp mobile application/ OR mobile app*.mp. or exp mobile application/ OR tablet computer*.mp. or exp tablet computer/ OR mobile health.mp. OR mHealth.mp. OR digital health.mp. OR online health monitoring.mp. or exp patient monitoring/ | 322,360 |
| #2 | Outcome –  Physical activity or Sedentary behaviour | physical activity.mp. or exp physical activity/ OR exp exercise/ or exercise.mp. OR aerobic exercise.mp. or exp aerobic exercise/ OR physical fitness.mp. or exp fitness/ OR exp fitness/ or fitness.mp. OR active lifestyle.mp. OR exercise capacity.mp. or exp exercise/ OR exercise therapy.mp. or exp kinesiotherapy/ OR physical exertion.mp. or exp exercise/ OR exp sedentary lifestyle/ or sedentar*.mp. OR exp physical inactivity/ or physical inactiv*.mp. OR sedentary behaviour.mp. or exp sedentary lifestyle/ OR sitting.mp. or exp sitting/ OR sit time.mp. OR screen time.mp. or exp screen time/ OR sport*.mp. or exp sport/ OR exp walk test/ or walk*.mp. OR movement.mp. or exp "movement (physiology)"/ OR healthy lifestyle.mp. or exp healthy lifestyle/ or exp health behavior/ or exp health promotion/ | 2,039,149 |
| #3 | Population –  Cardiovascular disease | cardiovascular disease.mp. or exp cardiovascular disease/ OR exp cardiovascular disease/ or cardiovascular.mp. OR cardiac rehabilitation.mp. or exp heart rehabilitation/ OR exp cardiac patient/ or cardiac.mp. OR coronary artery disease.mp. or exp coronary artery disease/ OR coronary heart disease.mp. or exp ischemic heart disease/ OR coronary disease.mp. or exp coronary artery disease/ OR exp hypertension/ or hypertension.mp. OR myocardial infarction.mp. or exp heart infarction/ OR stroke.mp. or exp cerebrovascular accident/ OR heart failure.mp. or exp heart failure/ OR arrhythmia.mp. or exp heart arrhythmia/ OR peripheral artery disease.mp. or exp peripheral occlusive artery disease/ OR cerebral vascular disease.mp. or exp cerebrovascular disease/ OR Heart disease.mp. or exp heart disease/ OR acute coronary syndrome.mp. or exp acute coronary syndrome/ OR secondary prevention.mp. or exp secondary prevention/ | 4,622,668 |
| #4 |  | #1 AND #2 AND #3 | 1,376 |
