# Supplementary material for: Smartphone applications for physical activity and sedentary behaviour change in people with cardiovascular disease: A systematic review and meta-analysis
Source: PLoS One. 2021 Oct 11;16(10):e0258460. doi: 10.1371/journal.pone.0258460 (PMC8504773; doi:10.1371/journal.pone.0258460)
Supplement: S2 File — (DOCX) [file pone.0258460.s006.docx]

**Supporting information 2 File: Moderate to vigorous intensity physical activity minutes per week subgroup analysis results**

**
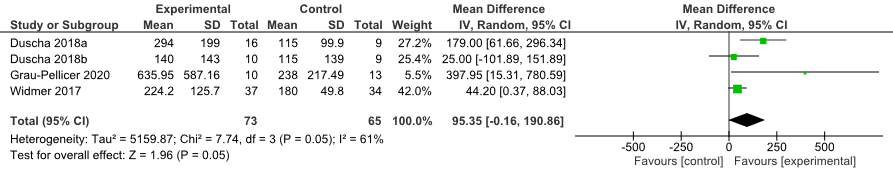
**

Fig a. Mean difference for studies with and intervention length ≤ 3-months and participant age > 60yrs


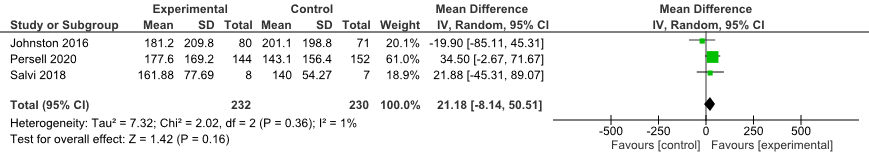


Fig b. Mean difference for studies with a mean participant age ≤ 60yrs


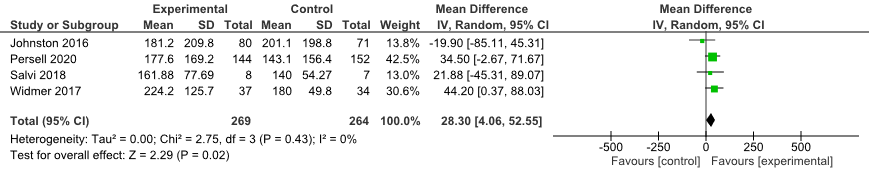


Fig c. Mean difference for interventions that only used a smartphone app


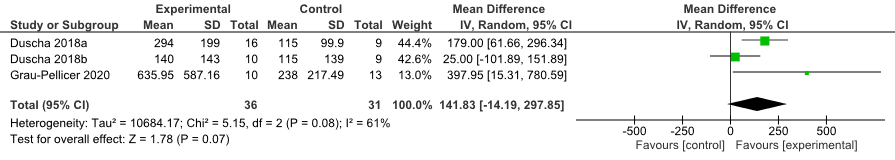


Fig d. Mean difference for interventions that used an activity tracker with the smartphone app


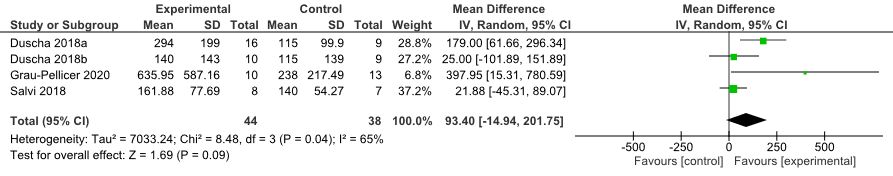


Fig e. Mean difference for interventions which targeted physical activity
